# Hospital length of stay prediction tools for all hospital admissions and general medicine populations: systematic review and meta-analysis

**DOI:** 10.3389/fmed.2023.1192969

**Published:** 2023-08-16

**Authors:** Swapna Gokhale, David Taylor, Jaskirath Gill, Yanan Hu, Nikolajs Zeps, Vincent Lequertier, Luis Prado, Helena Teede, Joanne Enticott

**Affiliations:** ^1^Monash Centre for Health Research and Implementation, Faculty of Medicine, Nursing, and Health Sciences, Monash University, Clayton, VIC, Australia; ^2^Eastern Health, Box Hill, VIC, Australia; ^3^Office of Research and Ethics, Eastern Health, Box Hill, VIC, Australia; ^4^Alfred Health, Melbourne, VIC, Australia; ^5^Monash Partners Academic Health Sciences Centre, Clayton, VIC, Australia; ^6^Eastern Health Clinical School, Monash University Faculty of Medicine, Nursing and Health Sciences, Clayton, VIC, Australia; ^7^Univ. Lyon, INSA Lyon, Univ Lyon 2, Université Claude Bernard Lyon 1, Lyon, France; ^8^Research on Healthcare Performance (RESHAPE), INSERM U1290, Université Claude Bernard Lyon 1, Lyon, France; ^9^Epworth Healthcare, Academic and Medical Services, Melbourne, VIC, Australia

**Keywords:** risk assessment/risk prediction tools/factors/methods, length of stay, regression, machine learning, medicine

## Abstract

**Background:**

Unwarranted extended length of stay (LOS) increases the risk of hospital-acquired complications, morbidity, and all-cause mortality and needs to be recognized and addressed proactively.

**Objective:**

This systematic review aimed to identify validated prediction variables and methods used in tools that predict the risk of prolonged LOS in all hospital admissions and specifically General Medicine (GenMed) admissions.

**Method:**

LOS prediction tools published since 2010 were identified in five major research databases. The main outcomes were model performance metrics, prediction variables, and level of validation. Meta-analysis was completed for validated models. The risk of bias was assessed using the PROBAST checklist.

**Results:**

Overall, 25 all admission studies and 14 GenMed studies were identified. Statistical and machine learning methods were used almost equally in both groups. Calibration metrics were reported infrequently, with only 2 of 39 studies performing external validation. Meta-analysis of all admissions validation studies revealed a 95% prediction interval for theta of 0.596 to 0.798 for the area under the curve. Important predictor categories were co-morbidity diagnoses and illness severity risk scores, demographics, and admission characteristics. Overall study quality was deemed low due to poor data processing and analysis reporting.

**Conclusion:**

To the best of our knowledge, this is the first systematic review assessing the quality of risk prediction models for hospital LOS in GenMed and all admissions groups. Notably, both machine learning and statistical modeling demonstrated good predictive performance, but models were infrequently externally validated and had poor overall study quality. Moving forward, a focus on quality methods by the adoption of existing guidelines and external validation is needed before clinical application.

**Systematic review registration:**

https://www.crd.york.ac.uk/PROSPERO/, identifier: CRD42021272198.

## Background and significance

Hospital inpatient and outpatient services make up the bulk of the health spending for all the Organization for Economic Co-operation and Development (OECD) countries (1). Australian health expenditure has increased by an average of 2.7% per year in the last 18–20 years, and the cost of hospital care accounted for 40% of the total, of which 61.7% was spent on acute admitted care (2, 3). In 2020–2021, the cost of acute admitted care was AUD33.8 billion, with the average cost per admitted acute care separation being $5,315 (4). Length of stay (LOS) in an acute hospital is a significant influencer of the cost of delivering hospital-based care and is a key measure of hospital performance according to the Australian Health Performance Framework (5). Extended LOS increases the risk of hospital-acquired complications (HACs) and impacts patient access and flow (6). A recent report showed up to a 3- to 4-fold variation in the average LOS in Australian hospitals (3) often due to a complex interaction of multiple factors, including some unrelated to the patient's condition. HACs similar to delirium can prolong hospital LOS by 6–7 days and increase mortality (7, 8). Reducing unwanted variation in LOS is essential in Australia and globally to ensure the sustainability of economically viable health services for the future.

To utilize healthcare resources efficiently, studies have been undertaken globally utilizing existing data and applying statistical techniques such as machine learning (ML), to develop and validate predictive models identifying patients at risk of extended LOS (9–13). Prior studies have investigated LOS prediction in disease-specific groups such as heart failure (14), cardiac surgery (15), thermal burns (16), or population-specific groups such as intensive care unit (ICU) and neonatal care (17, 18). Other recent reviews have looked at this outcome from a risk adjustment perspective (19) or a broad epidemiological perspective (20).

Prediction of risk of extended LOS in heterogenous populations such as all hospital admissions and General Medicine is common but lacks impact (20, 21). Accurate and timely risk prediction can enable targeted interventions to streamline care, reduce unwarranted extended LOS, and potentially impact system-level management of patient flow issues by providing high-level visibility of impending access issues and enabling proactive decision-making (2, 22). A review of the literature published in 2019 had examined methodologies applied to create LOS predictions. The authors found that approximately half of the included studies (36 of 74) did not restrict the studied population by diagnosis groups, and only a third had calculated the prediction at the time of admission or earlier (20). We aimed to extend this review by broadening the search, evaluating the risk of bias (ROB) (23) of the included studies, and adding data from the recent 2 years to capture the emerging Artificial Intelligence (AI/ML) approaches. This review aims to identify validated prediction variables and methods used in tools that predict the risk of extended LOS in all hospital admissions and specifically General Medicine admissions. This is needed to advance the evidence base required by healthcare administrators and planners on possible future predictive tools supporting efficient resource utilization and patient flow.

## Methods

“Prediction tools” or “tools” for this review can include any type of risk assessment tools/flags/factors or risk prediction models that used computerized statistical methods for predicting hospital LOS. This review was conducted according to the Preferred Reporting Items for Systematic Reviews and Meta-Analyses (PRISMA) guidelines (24). Protocol was registered on the International Prospective Register of Systematic Reviews (PROSPERO) (https://www.crd.york.ac.uk/PROSPERO/) (#CRD42021272198).

### Search strategy

We searched CINAHL, EMBASE, OVID MEDLINE, OVID EMCARE, and Cochrane systematically on 31 August 2021 and updated the search on 28 June 2023, using a predefined search strategy guided by our library scientist (VD), as shown in Supplementary Table S2. The primary concepts searched were “risk factors”, “statistical/prediction models”, and “Length of stay”. Considering the rapidly advancing field of health data analytics, we narrowed the search to only include English language articles, from OECD comparable countries and published after 2010. Reference lists of included publications were examined to identify any additional potential studies. A gray literature search using key terms was completed in Google and Google Scholar in a time-limited way (20 h over 4 weeks).

### Eligibility criteria

As shown in Supplementary Table S3, we included primary studies that reported LOS predictive tools for adults admitted to acute care hospitals that reported prediction metrics (25) to inform what works in LOS prediction methods and in what context. No limits on publication types were applied. We excluded studies looking at day procedures (LOS < 24 h) and those describing or including admissions to nursing homes, or subacute/rehabilitation facilities due to the difference in their operational structure and purpose, compared to the acute hospital setting.

Model for all admissions (mixed medical and surgical admissions) was the focus based on recent reports suggesting the positive impact of identifying and managing acuity on hospital resource utilization (26). We also studied the prediction tools for the General Medicine admissions (2, 3, 5) due to their high LOS variation, which is summarized in a separate section.

Studies that were not primary research, including conference abstracts, unpublished studies, book chapters, and review articles, were excluded. We also excluded reports focusing on condition/procedure-specific LOS tools such as burns, joint replacements, cardiology, cancer, maternity, and pediatric admissions and studies that did not assess LOS as an outcome.

No limits on publication types were applied. Once studies were highlighted for inclusion, the reference lists of included publications were manually searched for additional studies.

### Study screening and data extraction

Screening, full-text review, data extraction, and quality assessment were completed using the web-based data management platforms of Covidence (27) and EndNote X9.3.3 (Clairvate). Title, abstract, and full-text screening was conducted by two reviewers (SG and JG) who were responsible for selecting studies for inclusion. In case of discrepancies, consensus was reached via discussion. SG extracted data based on the CHARMS and TRIPOD checklist (28, 29) into a predefined data extraction table.

### Quality assessment

The risk of bias was assessed independently by two reviewers (SG and YH) based on PROBAST recommendations. Disagreement was resolved by consulting a third reviewer (JE). Using the PROBAST tool (30), studies were rated as low/moderate/high concern for bias and applicability in each of the four domains: participants, predictors, outcomes, and analysis (23, 29). We used guidance from the adaptation of the PROBAST tool for ML models (31).

### Data synthesis

The data items extracted for each included article are provided in Supplementary Table S4. Data sources were classified as (1) administrative/registry/claims, and (2) medical records and prediction modeling methods as classic statistical methods/ML/both. Model performance measures of discrimination and calibration were extracted and synthesized.

Discrimination measures, where possible, were presented as Area Under Receiver Operating Curve (AUROC) with a 95% confidence interval (CI) (21). We applied AUROC thresholds of 0.5 to suggest no discrimination (ability to identify patients with and without the risk under test), 0.7–0.8 as acceptable, 0.8–0.9 as excellent, and >0.9 as outstanding discrimination (32). Calibration was assessed using reported calibration plots, where available, or using calibration statistics (32, 33).

Predictor variables in the included LOS models were classified into categories adapted from the recent systematic review by Lequertier et al. (20), as shown in Supplementary Table S5. The level of validation (development with or without internal validation and/or external validation) was based on the PROBAST guideline (30).

### Meta-analysis

Meta-analysis of prediction models is challenging especially when models are specified differently and have heterogenous predictors and outcome definitions (34). Conversely, it is also valuable to understand the impact of the underlying variation in case mix and population characteristics on the prediction estimates (35). As such, we have presented a random-effects meta-analysis using restricted maximum likelihood estimation for external validation studies of LOS prediction models. As guided by recent literature on a meta-analysis of prediction model studies (36, 37), models having comparable outcome types (binary) and predictors were included, and we reported the 95% prediction interval of theta (21) to provide a range for the estimated performance of the model in a new population. Stata SE 17 was used for statistical analysis and calculation. When the standard error of AUROC was unreported, it was estimated using the method by Hanley and McNeil (38) and Kottas et al. (39). Heterogeneity was reported as *I*^2^ (40). The number of eligible validation studies was small, and hence further investigation of sources of heterogeneity was not possible.

### Publication bias

Forest plots showing effect sizes and confidence intervals were generated. Egger's regression was used for evaluating funnel plot asymmetry due to small-study effects (33, 41).

## Results

**Figure 1:**
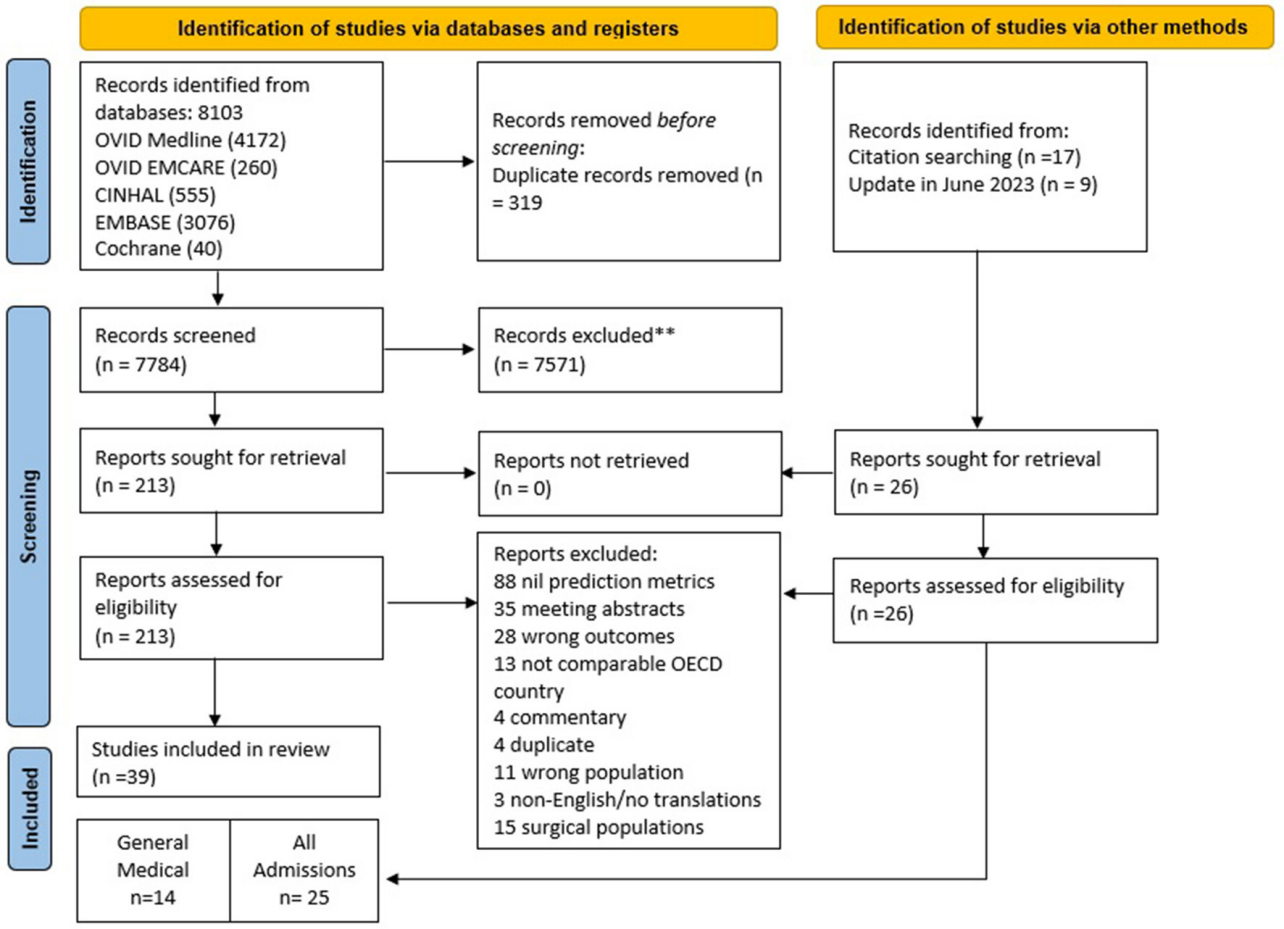
PRISMA flow diagram demonstrates the systematic review of the literature for hospital length of stay prediction tools. PRISMA, preferred reporting items for systematic reviews and meta-analyses; ^**^ based on exclusion criteria provided in Supplementary Table S3; OECD, organization for economic co-operation and development.

The search yielded 8,103 studies from OVID Medline (4,172), OVID Emcare (260), CINHAL (555), EMBASE (3-076), and Cochrane (40). Records were exported to Covidence, and 319 duplicates were removed. In total, 7,784 records were screened, which yielded 213 potential reports for full-text retrieval. Citation searching identified an additional 17 records which were assessed for eligibility. A recent update identified a further nine studies for full-text review. Following the full-text review, 39 were selected for inclusion based on the eligibility criteria: 14 reporting on GenMed populations and 25 on all admissions. PRISMA diagram illustrates the search in Figure 1. Study characteristics are summarized in Supplementary Table S6.

### All admissions prediction models

Of the 25 studies, the majority were published in the last 5 years, 11 were from the United States, six from the European Union, two from Australia, and one each from the United Kingdom, Canada, Japan, South Korea, Algeria, and Singapore. All studies were observational: two prospective and 22 retrospective, a single cross-sectional study. The median duration was 3.75 years (range 0.6–12) with a median sample size of 53,211 (range 332–42,896,026).

#### Data sources

There was greater use of medical records data (60%) compared to administrative data (40%). All studies collected data at and during admission (84%) or used data collected post-discharge in addition to admission data. LOS was predicted categorically in 64% or continuously in 28% of studies and both categorically and continuously in 8% of studies. The cut-off for defining prolonged LOS ranged from 5 to 14 days, and two studies used a predefined diagnosis-specific increase of LOS tertile as their cut-off.

#### Predictive modeling methods

The level of validation was low with only 2 of 25 reported validation studies (four models). Of the 45 models reported in 25 studies, classical statistical approaches accounted for just under half (44%), ML methods such as ridge regression, random forest, gradient boosting machine algorithms, and generalized linear models were used in 32%, and deep learning approaches (24%) included stacked recurrent neural network, channel-wise long short-term memory (LSTM), multi-modal deep learning, and ensemble-based neural networks. The greater prevalence of ML and deep learning approach in this group is likely to reflect the number and complexity of the variables and the large sample size used in these studies.

#### Analytical pipeline

The median number of predictors used was 18 (range 2–714). Inclusion of all candidate predictors in multivariable modeling was common (96%) without pre-selection of variables which was done in a single study (42). Feature/predictor selection methods during multivariable modeling were largely poorly reported in 76% of studies. When reported, AIC (43–45), recursive feature elimination (46), and full model approach (47, 48) were used for feature/predictor selection. Missing data were handled using imputation by various methods in 16% of studies but remained under-reported in the remaining studies (84%). Methods used to manage over-fitting and optimism were commonly used in 80% of studies. They included combinations of random split, *k*-fold cross-validation, bootstrapping, hyper-parameter tuning and selection and stochastic gradient descent techniques; and were not reported in 20% of studies. The more recent studies reported various hyperparameter optimisation methods such as Bayesian (49) and Gaussian (50)-based selection and tuning processes, gradient descent methods (51), and 10-fold cross-validation (52).

Table 1 and Supplementary Table S8 show the key information for all admission LOS prediction models included in the systematic review.

**Table 1:** All admission LOS prediction models included in the systematic review (*n* = 45).

**References**	**Type of final model**	**Outcome**	**Name of data analysis/modeling method used DD**	**AUROC values (95% CI)/C-statistics**	**Other prediction metrics**
Baek et al. (42) (1)	Internal validation	LOS Pred (continuous)	Multivariable logistic regression		MAE = 4.68
Baek et al. (42) (2)		LOS long-term (>30 days)	Random forest method (ML)		Accuracy: 97.32%
Bahrmann (53) (1)	Development	LOS (continuous)	Multivariable linear regression		Estimate: −0.58 (−1.0, −0.15) *p* = 0.009
Bahrmann (53) (2)					Estimate: 0.41 (0.02, 0.81) *p* = 0.041
Beaulieu-Jones (54) (1)	Development	LOS > 7 days	Stacked recurrent neural network [gated recurrent unit (GRU)]	0.82	
Beaulieu-Jones (54) (2)	Temporal validation			0.71	
Belderrar (55)	Internal validation	High hospital LOS outliers (geometric mean = 2 SD)	FRBFN (fuzzy radial basis function networks)		MMRE (*Z*-score): 2.13%
Chrusciel (56)	Development	LOS ≥ 7 days (structured data)	Random forest method (ML)		Accuracy: 74.1% • Precision: 74.2%
		LOS ≥ 7 days (unstructured data)			Accuracy: 75% • Precision: 75.7%
Gilbert et al. (57)	Internal validation	LOS > 10 days	Multivariable logistic regression	0.73	
Grampurohit et al. (58)	Development	LOS (continuous)	Ridge regression		MAE: 0.82131
Guerra et al. (43) (1)	Development	LOS ≥ 7 d	Cox proportional hazards regression model		HR = 0.60 (0.49–0.73) • AIC 6006
Guerra et al. (43) (2)					HR = 0.61 (0.52–0.73) • AIC 6019
Harutyunyan et al. (59) (1)	Internal validation	LOS > 7 days	Channel-wise LSTM + deep supervision	0.84	
Harutyunyan et al. (59) (2)		LOS (continuous)			MAE: 94.0 (93.6, 94.4)
Hilton et al. (49)	Internal validation	LOS > 5 days	Gradient boosting machine (GBM)-based methods	0.84	
Jaotombo et al. (52)	Development	LOS > 14 days	Gradient boosting machines (GBM)	0.81	
Lequertier et al. (20)	Internal validation	LOS 0–13 days • LOS > 13 days	Feed-forward neural network (FFNN) with embeddings		Accuracy: 73%
Levin et al. (47) (1)	Internal validation	LOS < 1 day (same-day discharge)	Supervised ML	0.72–0.78	
Levin et al. (47) (2)		LOS < 2 days (Next Day Discharge)	Supervised ML	0.70–0.80	
Liu (60) (1)	Development	LOS > 5 days	Multivariable logistic regression	0.81 (0.81–0.82)	
Liu (60) (2)	Development			0.90 (0.90–0.91)	
Liu (60) (3)	Development			0.94 (0.93–0.94)	
Liu (61)	Development	LOS (continuous)	OLS linear regression		Accuracy: 62.9%
Malone (62)	Internal validation	LOS (continuous) time series data only	Ridge regression		MAE: 2.956
Malone (62)		LOS (continuous) all data			MAE: 2.945
McAlister and van Walraven (48) (1)	External validation	LOS > 10 d	Multivariable logistic regression	0.705	
McAlister and van Walraven (48) (2)	External validation			0.723	
Monterde et al. (44) (1)	Development	LOS >14 days	Multivariable logistic regression	0.739 (0.734–0.743)	
Monterde et al. (44) (2)	Development			0.786 (0.782–0.790)	
Monterde et al. (44) (3)	Development			0.745 (0.740–0.750)	
Monterde et al. (44) (4)	Development			0.811 (0.806–0.815)	
Ossai et al. (46)	Development	Tertile for DRG	Machine learning: SMOTE + recursive feature elimination with cross-validation (RFECV) + extra tree classifier (ETC)		Accuracy 0.885 ± 0.063 • Precision 0.9 ± 0.052
Purushotham et al. (45)	Internal validation	LOS (continuous)	MMDL (multi-modal deep learning) using data in the first 24 h		MSE: 36,338.2015 ± 2,672.3832
Purushotham et al. (45)	Internal validation	LOS (continuous)	MMDL (multi-modal deep learning) using data in the first 48 h		MSE: 36,924.2312 ± 3,566.4318
Purushotham et al. (45)	Development	LOS (continuous)	MMDL (multi-modal deep learning) using data for the entire admission		MSE: 36,338.2015 ± 2,672.3832
Rajkomar et al. (50) (1)	Internal validation	LOS > 7 days Hospital A		0.86 (0.86–0.87)	
Rajkomar et al. (50) (2)	Internal validation	LOS > 7 days Hospital B	Deep learning	0.85 (0.85–0.86)	
Shin (63)	Internal validation	LOS (continuous)	GLM with gamma distribution		Explained variance: 0.088 (0.086–0.089)
Shukla (64)	Internal validation	LOS (continuous)	Interpolation and prediction network		Median absolute error: 2.862 ± 0.166 • Explained variance: 0.245 ± 0.019
Soong et al. (65) (1)	Development (elective)	Upper quartile of LOS specific to country	Multivariable logistic regression	0.73	
Soong et al. (65) (2)	Development (non-elective)		Multivariable logistic regression	0.65	
Soong et al. (65) (3)	External validation (elective)		Multivariable logistic regression	0.676	
Soong et al. (65) (4)	External validation (non-elective)		Multivariable logistic regression	0.677	
Xiongcai et al. (66)	Internal validation	LOS < 1 day (same-day discharge)	Machine learning	0.83	

Sn, sensitivity; Sp, specificity; MAPE, mean absolute percentage error; RMSE, root mean square error; SD, standard deviation; MAE, mean absolute error; AIC, Akaike's information criterion; HR, hazards ratio; OLS, ordinary least squares; GLM, generalized linear model.

#### Reported performance metrics and interpretation

**Figure 2:**
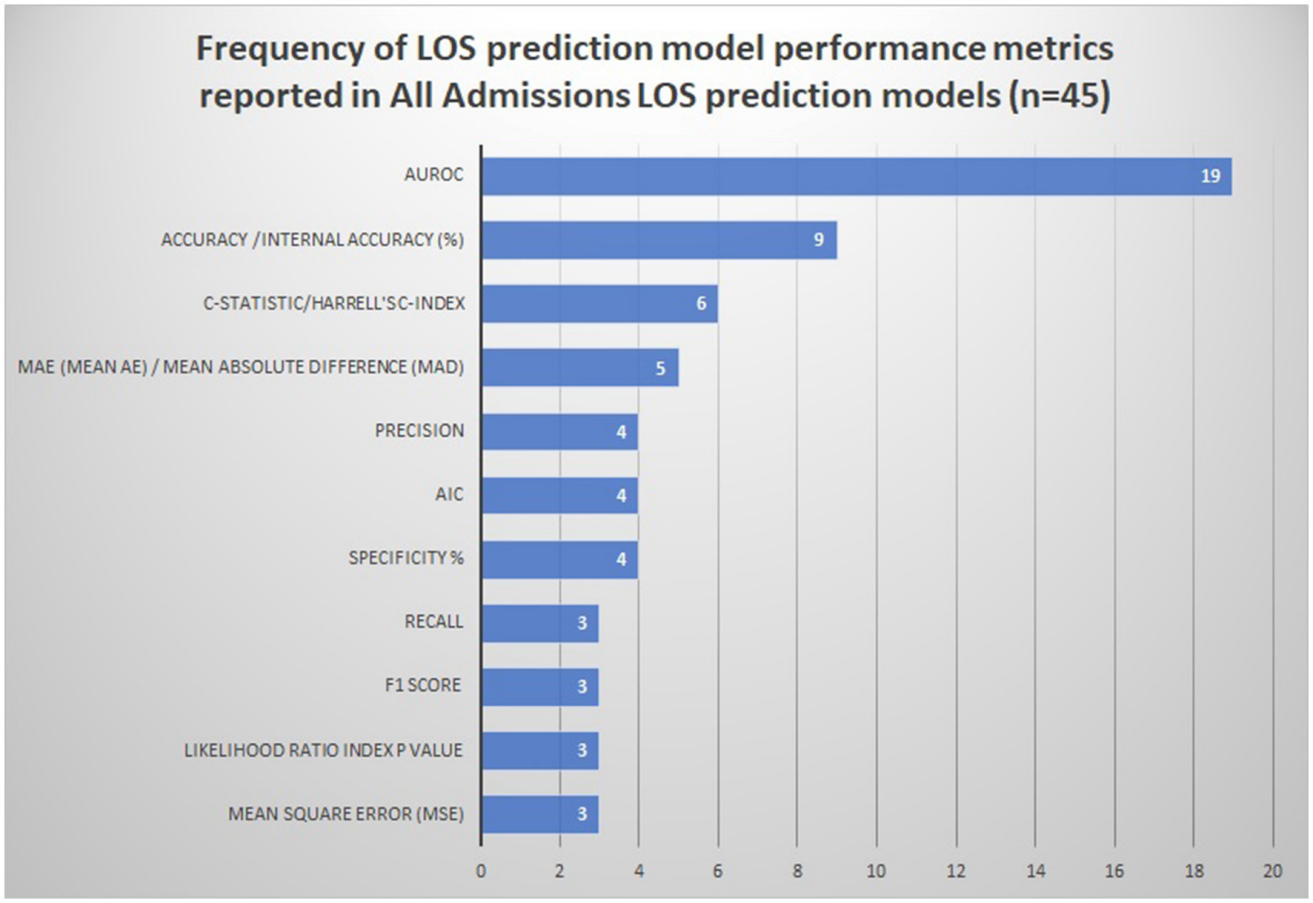
Frequency of LOS prediction model performance metrics reported in all admissions LOS prediction models (*n* = 45). AIC Akaike information criterion. The following performance metrics were used less than three times and are not represented in the figure: Pred/z-score/MMRE (mean magnitude of relative error), model adequacy/model fit *R*2/adjusted *R*-squared, Cohen's kappa, explained variance/Nagelkerke's *R*-squared, Brier score, and median AE (absolute error).

The frequency of the various reported model performance measures is summarized in Figure 2 and Supplementary Table S7.

#### Discrimination

AUROC was the most frequently reported metric of discrimination (42% models) outlined in Figure 2. The median values of AUROC were 0.7365 (range 0.63–0.832), indicating the fair-to-good discriminative ability of the majority of the models (67). Other discrimination metrics reported were accuracy (20%), C-statistic (13%), and mean absolute error (MAE) (11%).

#### Calibration

Calibration metrics (likelihood ratio index, HL goodness of fit, and calibration plots) were reported in only 20% of models. All the reported models appeared to be sufficiently calibrated.

Of the two studies reporting comprehensive performance measures, including calibration, discrimination, and overall accuracy measures, both Harutyunyan et al. (LOS>7 days) and Hilton et al. (LOS>5 days) demonstrated an excellent discriminative ability with AUROC of 0.84 (49, 59) with good calibration of models using ML/deep learning (recurrent neural networks, LSTM, and gradient boosting machines) and data from electronic medical records.

#### Predictors/variables

The most frequently used predictors and predictor categories are outlined in Table 2 and Supplementary Figure 1. Variable/feature importance was reported in half the studies using diverse association metrics such as hazard ratio, incident rate ratio, and estimates/regression coefficients making comparisons based on the strength of association of predictors imprecise.

**Table 2:** Most frequently used variables in risk prediction of prolonged LOS in all admissions (*n* = 25).

**Input variables (predictors)**	**Frequency of inclusion in LOS risk prediction studies (*****n*** = **25)**	
***Risk scores*** • **Illness severity scores** such as APR-DRG risk and APR-DRG severity • **Functional indices** such as Barthel's index (BI), hand grip strength (lowest three quartiles), and rehabilitation (mobility scale) • **Co-morbidity scores** such as CCI, Charlson age co-morbidity, Charlson co-morbidities, co-morbidity point score, Elixhauser co-morbidity score, Gagne's co-morbidities, and Queralt index • **Frailty scores** such as Dr. Foster Frailty Index and Hospital Frailty Risk Score (HFRS) • **Neuro-cognitive screens** such as GCS and its variations and triage scores • **Nutritional risk scores** such as MUST, NRS-2002, and PG-SGA	17	68%
**Demographic and anthropometric variables** • Age/sex • BMI • Caregivers • Clinical flags (correlates of psycho-social determinants) • Ethnicity: race (white vs. other) • Height/weight • Language • Marital status • Religion • Socioeconomic index	17	68%
**Admission characteristics** • Admission month/admission shift/admission source/admission type • Care units/hospital service/transfer frequency • Day of week time of day • Entry date and time • Mode of entry/mode of arrival to ED • Discharge date and time/discharge location • Early admission to ICU • Temporal variables: elapsed LOS (current admission)/last admission LOS/no. of days since last admission/total days in hospital in last 12/12 • First procedure on admission/medical procedures/interventions/procedural terminology	15	60%
**Physical examination (biological and physiological parameters)** • Observations: capillary refill rate, chart events, diastolic blood pressure, fraction of inspired oxygen, heart rate, mean blood pressure monitoring outputs, oxygen saturation, respiratory rate, temperature, systolic blood pressure • Lab tests: bilirubin, Glucose, ph, K, Na, serum bicarb level, serum urea, nitrogen level, WBC count • Laboratory acute physiology score • Number of micro labs/number of lab tests/consults/diagnostics (count of tests) • Imaging reports • Days since the last event (lab test, etc.)	10	40%
**Diagnoses (primary/secondary including co-morbidities) and procedure types**. • Principal diagnoses or admission diagnoses such as AIDS, blood cancers, mental co-morbidity, and metastatic cancer • Associated diagnoses • Number of diagnoses on admission	10	40%
**Administrative** • Administrative charge codes for all actions taken from a presentation at the hospital until the end of the first calendar day of admission • Insurance type	10	40%
**Medications** • 24 h medications • IV meds • Medications (count of meds Oral/IV) • Non-IV meds	4	16%
**Documentation and clinical notes** Data from Electronic medical record systems like CareVue and Meta-Vision including • observations • imaging • lab events • medication-related order entries • microbiology events • discharge summary	5	20%
**Healthcare professional characteristics** • Admitting physician speciality • Admitting unit/location	3	12%
**Hospital characteristics** • Type of hospital/center	2	8%

APR-DRG, all patients refined diagnosis related groups; CCI, charlson co-morbidity index; GCS, glasgow coma scale; MUST, malnutrition universal screening tool; NRS, nutrition risk screening; PG-SGA, patient-generated subjective global assessment.

The top three predictor categories used were risk scores (68%), demographic and anthropometric variables (68%), and admission characteristics (60%). Risk scores included illness severity scores, functional indices, co-morbidity scores, and neurocognitive screening tools. A wide range of demographic variables representing the social determinants of health (SDOH) such as ethnicity, socioeconomic index, anthropometric characteristics, and marital status were used frequently. Admission characteristics, such as admission source, day/month of admission, need for ICU admission, admitting unit, procedure type, time and length of last admission, elapsed LOS, and discharge/transfer destination, were used widely, possibly owing to the predominant use of medical record data sources and ongoing data collection throughout the admission period. Many studies using electronic medical records used information about the number of tests, consults, assessments, medication, and investigations as proxy indicators of extended stay rather than the actual results of these events (47, 51, 58, 66, 68).

Physical examination parameters and diagnostic and administrative variables were included in 40% of studies, while documentation and clinical notes, medications, health professional characteristics, and hospital characteristics were included less frequently. Admission diagnoses such as cancer and mental health conditions were noted as important features having an impact on LOS.

#### Quality assessment

The quality assessment of the included studies is outlined in Table 3. Although many retrospective studies were done using secondary data sources, most were deemed to be from high-quality databases with evident reporting standards.

**Table 3:** Risk of bias assessment of all admissions studies using PROBAST tool (*n* = 25).

	**Type of prediction model**	**Participant risk of bias**	**Predictor risk of bias**	**Outcome risk of bias**	**Analysis of risk of bias**	**Overall risk of bias**
Baek et al. (42)	Development	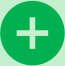	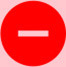	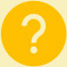	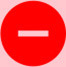	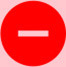
Bahrmann (53)	Development	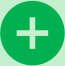	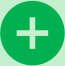	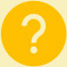	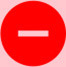	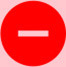
Beaulieu-Jones (54)	Validation	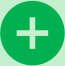	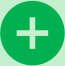	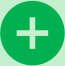	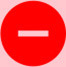	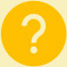
Belderrar (55)	Development	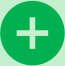	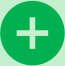	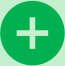	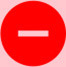	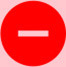
Chrusciel (56)	Development	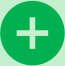	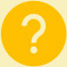	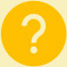	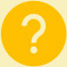	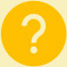
Gilbert et al. (57)	Development	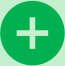	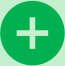	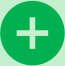	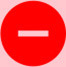	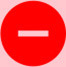
Grampurohit et al. (58)	Development	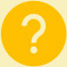	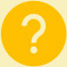	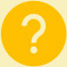	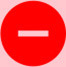	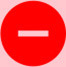
Guerra et al. (43)	Development	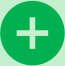	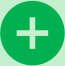	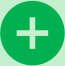	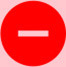	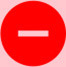
Harutyunyan et al. (59)	Development	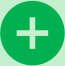	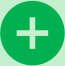	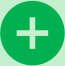	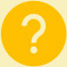	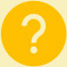
Hilton et al. (49)	Development	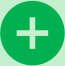	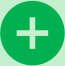	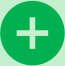	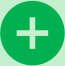	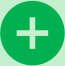
Jaotombo et al. (52)	Development	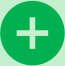	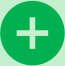	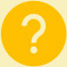	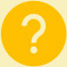	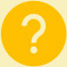
Lequertier et al. (20)	Development	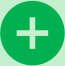	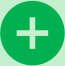	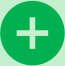	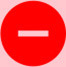	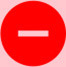
Levin et al. (47)	Development	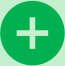	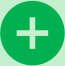	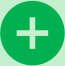	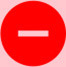	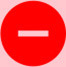
Liu (61)	Development	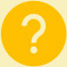	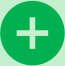	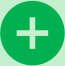	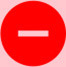	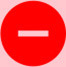
Liu (60)	Development	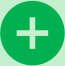	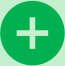	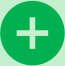	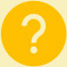	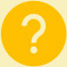
Malone (62)	Development	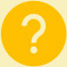	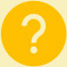	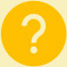	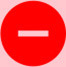	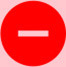
McAlister and van Walraven (48)	Validation	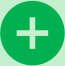	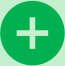	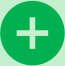	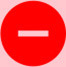	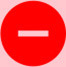
Monterde et al. (44)	Development	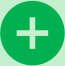	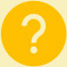	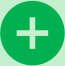	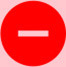	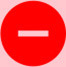
Ossai et al. (46)	Development	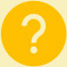	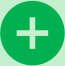	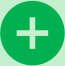	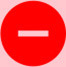	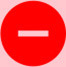
Purushotham et al. (45)	Development	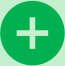	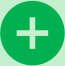	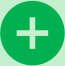	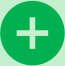	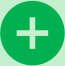
Rajkomar et al. (50)	Development	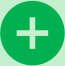	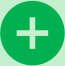	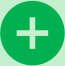	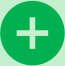	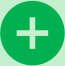
Shin (63)	Development	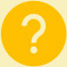	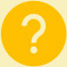	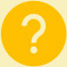	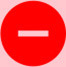	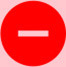
Shukla (64)	Development	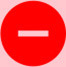	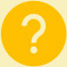	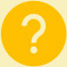	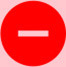	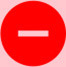
Soong et al. (65)	Validation	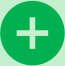	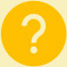	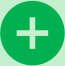	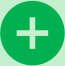	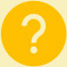
Xiongcai et al. (66)	Development	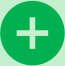	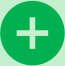	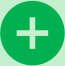	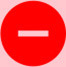	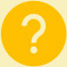

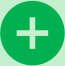
, low ROB; 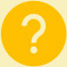, unclear ROB; 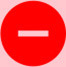, high ROB.

Dev, development only (includes models with internal validation), Val, studies with external validation.

Of the 25 studies, the majority of the studies were at a low ROB in domains of participants (76%), predictors (72%), and outcome (68%) domains, implying an overall low concern for applicability. Studies at moderate-to-high ROB in these domains demonstrated unclear reporting of data source quality, availability of predictors during implementation, determination, definition, and consistency of outcomes, and inappropriate participant inclusion/exclusion.

Quality assessment of analysis methods showed 68% were at high, and 16% at moderate or low risk of bias. Limitations in the model analysis and methodology reporting in high-risk studies included a lack of comprehensive reporting of model performance measures (no calibration measures), overfitting and optimism, missing data, and handling of data complexity, potentially implying poor adoption/awareness of the TRIPOD reporting guideline (29).

#### Meta-analysis

**Figure 3:**
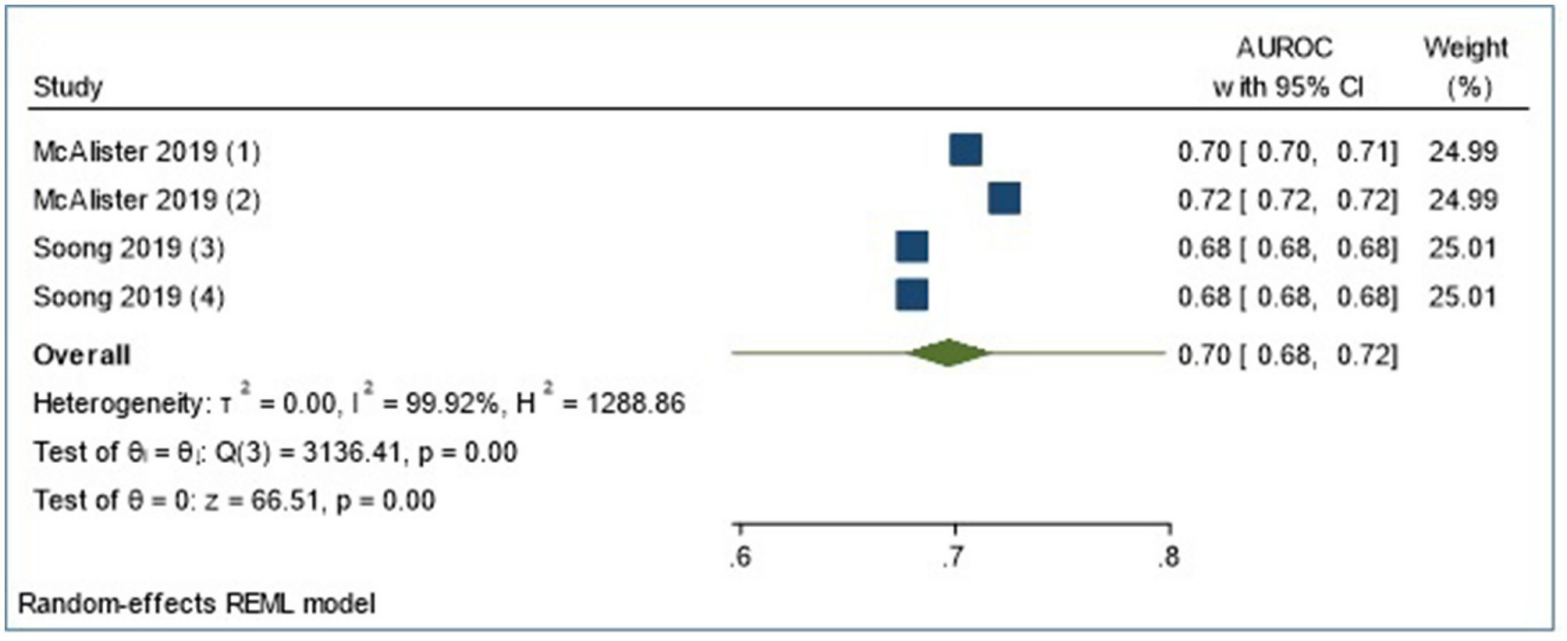
Meta-analyses of four externally validated models for LOS prediction in all admissions group (*n* = 4).

We conducted a meta-analysis of four LOS validation models that used Frailty Risk Scoring tools using administrative data [Hospital frailty risk score (48) and Global frailty score (65)] to predict LOS using logistic regression analysis. The meta-analysis reports a 95% prediction interval [shown in Figure 3 (forest plots), Table 4], to account for varying model performance due to differences in case mix and other study-level factors (21). The random-effects meta-analysis showed a 95% prediction interval for theta of 0.596, 0.798 (*I*^2^ = 99.92%). Sources of heterogeneity were not explored further statistically due to the small sample size. However, Supplementary Table 13 outlines the differences in study populations and characteristics.

**Table 4:** Meta-analysis summary of four externally validated models for LOS prediction in all admissions group.

**Meta-analysis summary**	
Number of studies = 4	
**Random-effects mode**	**Heterogeneity**
Method: REML	tau2 = 0.0004 *I*^2^ (%) = 99.92 H2 = 1,288.86
**95% prediction interval for theta** **=** **(0.596, 0.798)**	
Test of theta = 0: *z* = 66.51 Prob > |*z*| = 0.0000	
Test of homogeneity: Q = chi2(3) = 3,136.41 Prob > Q = 0.0000	

#### Publication bias

We observed no small-study effects on statistical testing (Egger's test *p* < 0.001) shown in Supplementary Table S11. In combination with the visual inspection of the funnel plots, we observed no publication bias in our included studies.

### General medicine prediction models

The majority of the studies in this subgroup came from Europe (nine of 14) and the rest from the United States, Australia, and Japan (3, 1, and 1, respectively). The median study duration was 2.9 years (range 0.2–12) with a median sample size of 19,095 (range 33–2,997,249) and the predominant use of administrative data (64%). Timing of prediction in most studies (13 of 14) was on admission with a large range of prolonged LOS cut-offs used (3–30 days).

#### Predictive modeling methods

There were no externally validated models in 30 models reported in 14 studies. Overall, 56% used classical statistical approaches such as multivariable logistic (*n* = 14) and Cox/Poisson (*n* = 3) regression. The rest were ML (37%) and deep learning (artificial neural network) (7%) models. Supervised ML methods used commonly were bagged regression trees (*n* = 3), random forest (*n* = 4), linear support vector machine (SVM) + Chi-square filtering method with synthetic minority over-sampling technique (SMOTE) (*n* = 3), and one decision tree (CHAID) model. Binary outcome modeling was more common (90% of models). AUROC was the most frequently reported metric of discrimination (46%) as outlined in Supplementary Figure 2 followed by sensitivity, specificity, and C-statistic.

#### Analytical pipeline

The median number of predictors used was 12 (5-1001). Most studies (64%) included all candidate predictors in multivariable modeling and pre-selection of variables based on univariable analysis was noted in 35% of studies. Feature/predictor selection methods during multivariable modeling and missing data were poorly reported. In the remaining studies (45, 69–72) *p*-value thresholds were used for feature/predictor selection, and patients with missing data were excluded (73–75). Methods used to manage overfitting and optimism were used frequently (64%) and included combinations of random split, *k*-fold cross-validation, bootstrapping, and sensitivity analysis.

#### Predictor variables

Frequently used variable predictor categories are shown in Supplementary Figure 1 and Supplementary Table 8. Predictor categories such as risk scores (86%), diagnoses (primary/secondary including co-morbidities) (79%), and demographic and anthropometric variables (71%) were used most frequently. Commonly used risk scoring tools were illness severity scores/index (71), Charlson Co-morbidity Index and Manchester triage scores (70), Brief Geriatric Assessment tool (74), Exton smith scale (pressure injury risk), ADL score and nutritional risk tools (76), and COMPRI (care COMplexity PRediction Instrument) (77). Cardiovascular, respiratory, gastrointestinal, and neurological diagnostic groups were noted as significant predictors, in addition to demographic characteristics such as age, sex, and living situation. Physical/laboratory parameters (43%) such as serum markers, routine observations including oxygen requirements, medication variables such as >5 drugs/day (36%), and admission characteristics (14%) such as day/month of admission, elapsed LOS, and discharge destination were also included in prediction models albeit less frequently. The predominant use of diagnostic categories in this group emphasizes the importance of clinical presentation in General Medicine admissions and diagnostic complexity reflecting acuity and by proxy LOS.

#### Quality assessment

Risk of bias (ROB) was low in the domains of predictors and outcome assessment of all studies (Supplementary Table 12). In total, 28% of studies were found to have a moderate-to-high bias in the participant selection domain due to unclear data source information. Bias was also noted to be high in the analysis of all included studies. The commonly observed pitfalls were a lack of comprehensive reporting of model performance measures (no calibration measures) (78%), overfitting and optimism (35%), missing data (85%), and handling of data complexity (71%). As a result, the overall ROB for all included General Medicine studies was high suggesting that results should be interpreted and translated cautiously.

## Discussion

This systematic review of risk prediction models for prolonged LOS in all admissions and General Medicine admissions showed a sharp increase in reporting of LOS prediction studies since 2018 with the widespread use of ML methods. Most models calculated the risk on admission. Reported prediction models showed good discriminative ability; however, they lacked calibration information, limiting impact assessment. Only four external validation models were reported with extensive use of electronic medical records and ML and AI methods. Overall, the study reporting was poor, especially for model analysis and performance, impacting the ability to assess the model quality and potential for translation into practice. In addition to detailed reporting aligning with guidelines such as TRIPOD and PROBAST, the high-quality studies had large sample sizes and reliable data sources and used retrospective data. A meta-analysis demonstrated prediction intervals in the moderate-to-good discrimination range, demonstrating that these macro-level algorithms may have some utility for identifying inpatients at risk of prolonged LOS.

Observations about a shortage of external validation studies have been noted by other researchers (78–80). Underreporting of external validation studies that often perform poorly may be contributing to this observation (80, 81). Another factor may be the lack of consistency in the predictor variables used in the various LOS models. Consensus on a consistent set of predictor variables could assist the ability of researchers across the world to conduct external validations and work toward establishing transportable models predicting the risk of prolonged LOS. Increasing age, presence of multiple co-morbidities (assessed via diagnoses or risk scores), illness severity (assessed using risk scores or proxy indicators such as number of medications), and admission characteristics such as type, source, and day of admission were used most frequently in the GenMed admissions. In addition to these, all admissions models predominantly included physiological measurements (such as BP and oxygen saturation) and functional independence measures (risk scores or demographic variables such as living situation). The extensive use of non-clinical features may suggest that systemic and environmental factors have a considerable role alongside clinical factors in the prediction of LOS in heterogenous populations.

Literature about procedure-specific prediction models with good prediction accuracy (82, 83) is abundant, with models primarily predicting clinical outcomes such as 30-day mortality and postoperative pain. LOS prediction models for surgical populations have been analyzed and published in a separate manuscript (84). LOS predictions are considered to have a dual benefit in being a proxy measure of clinical outcomes as well as hospital efficiency (1). As such, population-based LOS predictions are key enablers of organizational resource planning as well as the daily access and flow issues managed by the frontline staff. Hence, the purpose of prediction should guide the choice of procedure-specific vs. population-specific models.

SDOHs are also associated with health outcomes such as longer acute LOS (85, 86). Factors such as socioeconomic index, residential postcode, cohabitation status, and level of education are often considered a proxy for SDOH and can be extracted from routinely collected data. Only two studies (47, 57), in this review, explicitly used these factors over and above the standard demographic variables of age, gender, ethnicity, and marital status. Levin et al. included predictors such as addiction treatment medications, psychotherapeutics, case management and social work consults, and clinical flags of substance abuse, which were correlates of SDOH. Notably, only seven of 39 studies clearly indicated the inclusion of other socioeconomic variables such as ethnicity, race, religion, language, or marital status. This could potentially be a limitation of the data sources used or the capability for data linkage with other data sources which could provide this rich detail to the data. Future models could benefit from the inclusion of reliable indicators of SDOH to identify cases where prolonged LOS risk may be more ambiguous.

Clinical implementation and deployment of LOS prediction models continue to be a challenge despite extensive efforts in the development of such models (87–89). Low digital literacy levels, serious technological debt in healthcare infrastructure systems, and issues with the reliability of data and interoperability have been widely cited in the literature as potential roadblocks to the implementation of such predictive analytical decision support. In addition, successful implementation strategies must consider the existing workflows and clinician perspectives on the utility and value of these predictive algorithms. As such, co-design and coproduction with end-users is crucial to embed these tools as an integrated legacy framework, for future use by the health service. Furthermore, in this process, external validations must be conducted in a large number of settings to show all stakeholders, including clinicians, administrations, and patients, that this type of decision support can add value and is trustworthy.

### Strengths and limitations

The validated PROBAST quality assessment of the included studies was a strength of our review. It revealed a significant gap in the adoption of TRIPOD guidelines for prediction modeling studies, presenting evidence of moderate-to-high ROB. Poor reporting impacts implementation feasibility and external validation of existing prediction models. Many recent publications have implored the research community to attempt external validation before developing new models while accepting the evident challenges in reporting and reproducibility (80, 90). This review further strengthens this imperative to improve the reporting in prognostic prediction modeling studies in LOS.

The majority of the data sources in our systematic review were classified as secondary data sources. As per the PROBAST tool recommendation, secondary data sources are considered as high ROB due to a lack of data collection protocols, increasing the uncertainty about data validity (91) and limiting generalizability. Secondary data use is critical for long-term real-world evaluation of health interventions, system efficacy, and continuous improvement and monitoring of health service delivery (92). Transparent reporting of data quality issues such as missingness, inaccuracy, and inconsistency can assist in providing some reassurance that routinely collected data can be used as a strategic resource for research to improve health system efficiencies and effectiveness (91, 93, 94). We suggest that data hubs and repositories adopt evidence-based standardized frameworks to guide their data governance and evaluation practices (92, 95) to ensure transferability and generalization of results of secondary analysis of routinely collected health data.

### Broad recommendations

Future studies should (1) validate the prediction models on prospective data to enable near real-time LOS risk prediction and attempt external validation of existing models to test implementation feasibility, (2) use appropriate guidelines (23, 29) to report prediction study findings, (3) utilize data available on and within 24 h of admission to enable prognostic prediction and proactive interventions, and (4) include variables and assessments that are available from routinely collected data to reduce the administrative burden on frontline clinicians.

## Conclusion

To the best of our knowledge, this is the first systematic review assessing the quality of risk prediction models for prolonged LOS in All Admissions and GenMed studies. Overall, LOS risk prediction models appear to show an acceptable-to-good ability to discriminate, however, transparent reporting and external validations are now required for potential benefits of such macro-level prediction tools to be implemented inside hospitals to assist with early identification of inpatients at risk of a prolonged LOS.

## Data availability statement

The original contributions presented in the study are included in the article/Supplementary material, further inquiries can be directed to the corresponding author/s.

## Author contributions

SG designed the study, conducted analyses, literature screening, data extraction, and wrote this manuscript. JE, DT, NZ, and HT contributed to the study conceptualization, development of the methodological approach, and validation of analyses. JG and YH participated in screening, full-text review, data extraction validation, and bias assessment. VL provided technical guidance for the extraction and interpretation of results. All authors revised the manuscript. All authors contributed to the article and approved the submitted version.
