# Supplementary material for: Hospital length of stay prediction tools for all hospital admissions and general medicine populations: systematic review and meta-analysis
Source: Front Med (Lausanne). 2023 Aug 16;10:1192969. doi: 10.3389/fmed.2023.1192969 (PMC10469540; doi:10.3389/fmed.2023.1192969)
Supplement: Supplementary file 1 [file Data_Sheet_1.pdf]

Supplementary file:

Hospital Length of Stay Prediction Tools for All Hospital admissions and General Medical Populations: Systematic Review and Meta-Analysis

Table S1 PRISMA checklist

Section and Topic	Item #	Checklist item	Location where item is reported (section)
**TITLE**			
Title	1	Identify the report as a systematic review.	Pg 1
**ABSTRACT**			
Abstract	2	See the PRISMA 2020 for Abstracts checklist.	Pg 1
**INTRODUCTION**			
Rationale	3	Describe the rationale for the review in the context of existing knowledge.	Pg 2
Objectives	4	Provide an explicit statement of the objective(s) or question(s) the review addresses.	Pg 2
**METHODS**			
Eligibility criteria	5	Specify the inclusion and exclusion criteria for the review and how studies were grouped for the syntheses.	Pg 2-3
Information sources	6	Specify all databases, registers, websites, organisations, reference lists and other sources searched or consulted to identify studies. Specify the date when each source was last searched or consulted.	Pg 2-3
Search strategy	7	Present the full search strategies for all databases, registers and websites, including any filters and limits used.	Pg 2-3
Selection process	8	Specify the methods used to decide whether a study met the inclusion criteria of the review, including how many reviewers screened each record and each report retrieved, whether they worked independently, and if applicable, details of automation tools used in the process.	Pg 2-3
Data collection process	9	Specify the methods used to collect data from reports, including how many reviewers collected data from each report, whether they worked independently, any processes for obtaining or confirming data from study investigators, and if applicable, details of automation tools used in the process.	Pg 2-3
Data items	10a	List and define all outcomes for which data were sought. Specify whether all results that were compatible with each outcome domain in each study were sought (e.g. for all measures, time points, analyses), and if not, the methods used to decide which results to collect.	Supplement Table 4
	10b	List and define all other variables for which data were sought (e.g. participant and intervention characteristics, funding sources). Describe any assumptions made about any missing or unclear information.	Supplement Table 3
Study risk of bias assessment	11	Specify the methods used to assess risk of bias in the included studies, including details of the tool(s) used, how many reviewers assessed each study and whether they worked independently, and if applicable, details of automation tools used in the process.	Pg 2-3
Effect measures	12	Specify for each outcome the effect measure(s) (e.g. risk ratio, mean difference) used in the synthesis or presentation of results.	Pg 2-3
Synthesis	13a	Describe the processes used to decide which studies were eligible for each synthesis	Pg 2-3

Section and Topic	Item #	Checklist item	Location where item is reported (section)
methods		(e.g. tabulating the study intervention characteristics and comparing against the planned groups for each synthesis (item #5)).	
	13b	Describe any methods required to prepare the data for presentation or synthesis, such as handling of missing summary statistics, or data conversions.	Pg 2-3
	13c	Describe any methods used to tabulate or visually display results of individual studies and syntheses.	Pg 2-3
	13d	Describe any methods used to synthesize results and provide a rationale for the choice(s). If meta-analysis was performed, describe the model(s), method(s) to identify the presence and extent of statistical heterogeneity, and software package(s) used.	Pg 2-3
	13e	Describe any methods used to explore possible causes of heterogeneity among study results (e.g. subgroup analysis, meta-regression).	Pg 2-3
	13f	Describe any sensitivity analyses conducted to assess robustness of the synthesized results.	Pg 2-3
Reporting bias assessment	14	Describe any methods used to assess risk of bias due to missing results in a synthesis (arising from reporting biases).	Pg 2-3
Certainty assessment	15	Describe any methods used to assess certainty (or confidence) in the body of evidence for an outcome.	Pg 2-3
**RESULTS**			
Study selection	16a	Describe the results of the search and selection process, from the number of records identified in the search to the number of studies included in the review, ideally using a flow diagram.	Pg 3-11
	16b	Cite studies that might appear to meet the inclusion criteria, but which were excluded, and explain why they were excluded.	PRISMA flowchart Fig 1
Study characteristics	17	Cite each included study and present its characteristics.	Table 1 and Supplement table 6
Risk of bias in studies	18	Present assessments of risk of bias for each included study.	Pg 3-11
Results of individual studies	19	For all outcomes, present, for each study: (a) summary statistics for each group (where appropriate) and (b) an effect estimate and its precision (e.g. confidence/credible interval), ideally using structured tables or plots.	Supplement table 4
Results of syntheses	20a	For each synthesis, briefly summarise the characteristics and risk of bias among contributing studies.	Pg 3-11
	20b	Present results of all statistical syntheses conducted. If meta-analysis was done, present for each the summary estimate and its precision (e.g. confidence/credible interval) and measures of statistical heterogeneity. If comparing groups, describe the direction of the effect.	Pg 3-11
	20c	Present results of all investigations of possible causes of heterogeneity among study results.	Pg 3-11
	20d	Present results of all sensitivity analyses conducted to assess the robustness of the synthesized results.	n/a

Section and Topic	Item #	Checklist item	Location where item is reported (section)
Reporting biases	21	Present assessments of risk of bias due to missing results (arising from reporting biases) for each synthesis assessed.	n/a
Certainty of evidence	22	Present assessments of certainty (or confidence) in the body of evidence for each outcome assessed.	n/a
**DISCUSSION**			
Discussion	23a	Provide a general interpretation of the results in the context of other evidence.	Pg 11-12
	23b	Discuss any limitations of the evidence included in the review.	Pg 11-12
	23c	Discuss any limitations of the review processes used.	Pg 11-12
	23d	Discuss implications of the results for practice, policy, and future research.	Pg 11-12
**OTHER INFORMATION**			
Registration and protocol	24a	Provide registration information for the review, including register name and registration number, or state that the review was not registered.	Pg 1
	24b	Indicate where the review protocol can be accessed, or state that a protocol was not prepared.	Pg 1
	24c	Describe and explain any amendments to information provided at registration or in the protocol.	n/a
Support	25	Describe sources of financial or non-financial support for the review, and the role of the funders or sponsors in the review.	Pg 12-13
Competing interests	26	Declare any competing interests of review authors.	Pg 12-13
Availability of data, code and other materials	27	Report which of the following are publicly available and where they can be found template data collection forms; data extracted from included studies; data used for all analyses; analytic code; any other materials used in the review.	supplement

Table 2: Search strategy

Ovid MEDLINE(R) and Epub Ahead of Print, In-Process, In-Data-Review & Other Non-Indexed Citations, Daily and Versions 1946 to March 21, 2022	Embase Classic+Embase 1947 to 2022 March 21	Cochrane	CINHAL

1 Risk Assessment/ 297904<br>2 (risk adj1 (assess* or analys* or adjust*)).ti,ab,mp. 360526<br>3 "Severity of Illness Index"/ 266438<br>4 "Predictive Value of Tests"/ 220311<br>5 Risk Adjustment/ 4061<br>6 risk factors/ 915390<br>7 risk predict* factor*.ti,ab,mp. 30<br>8 Electronic Health Records/ 24661<br>9 Electronic Health Record*.ti,ab,mp. 33114<br>10 electronic medical record*.ti,ab,mp. 17689<br>11 electronic patient record*.ti,ab,mp. 2132<br>12 1 or 2 or 3 or 4 or 5 or 6 or 7 or 8 or 9 or 10 or 11 1573324<br>13 Regression Analysis/ 133531<br>14 exp Artificial Intelligence/ 139940<br>15 machine learning.ti,ab,mp. 43838<br>16 "Models, Statistical"/ 97702<br>17 Data Mining/ 10062<br>18 Logistic Models/ 149901<br>19 Computer Simulation/ 203426<br>20 computer simulation.ti,ab,mp. 207350<br>21 predict*.ti. 326499<br>22 (risk predict* adj1 (tool* or model*)).ti,ab,mp. 3612<br>23 (risk adjust* adj1 (tool* or model*)).ti,ab,mp. 758<br>24 13 or 14 or 15 or 16 or 17 or 18 or 19 or 20 or 21 or 22 or 23 967880<br>25 exp "Length of Stay"/ 98712<br>26 ((unplanned or unexpected or extended) adj4 stay*).ti,ab. 1411<br>27 (care adj1 days).ti,ab. 236<br>28 (length* adj2 stay*).ti,mp.	1. risk assessment/<br>2. (risk adj1 (assess* or analys* or adjust*)).ti,ab,mp.<br>3. "Severity of Illness Index"/<br>4. "Predictive value of tests"/<br>5. "prediction and forecasting"/<br>6. risk factors/<br>7. risk predict* factor*.ti,ab,mp.<br>8. Electronic Health Records/<br>9. medical record/ or electronic patient record/ or electronic medical record/<br>10. electronic medical record*.ti,ab,mp.<br>11. electronic patient record*.ti,ab,mp.<br>12. 1 or 2 or 3 or 4 or 5 or 6 or 7 or 8 or 9 or 10 or 11<br>13. regression analysis/<br>14. artificial intelligence/<br>15. machine learning/<br>16. statistical model/<br>17. Data Mining/<br>18. computer simulation/<br>19. computer simulation.ti,ab.mp<br>20. predict*.ti.<br>21. (risk predict* adj1 (tool* or model*)).ti,ab,mp.<br>22. (risk adjust* adj1 (tool* or model*)).ti,ab,mp.<br>23. 13 or 14 or 15 or 16 or 17 or 18 or 19 or 20 or 21 or 22<br>24. exp "length of stay"/<br>25. ((unplanned or unexpected or extended) adj4 stay*).ti,ab.<br>26. (care adj1 days).ti,ab.<br>27. (length* adj2 stay*).ti,mp.<br>28. 24 or 25 or 26 or 27<br>29. 12 and 23 and 28	MeSH descriptor: [Risk Assessment] this term only<br>(risk Near/1 (assess* or analys* or adjust*)):ti,ab,kw<br>MeSH descriptor: [Severity of Illness Index] this term only<br>MeSH descriptor: [Predictive Value of Tests] this term only<br>MeSH descriptor: [Risk Adjustment] this term only<br>MeSH descriptor: [Risk Factors] this term only<br>(risk predict* factor*):ti,ab,kw<br>MeSH descriptor: [Electronic Health Records] this term only<br>(electronic (health or medical or patient) record*):ti,ab,kw<br>#1 or #2 or #3 or #4 or #5 or #6 or #7 or #8 or #9<br>MeSH descriptor: [Regression Analysis] this term only<br>MeSH descriptor: [Artificial Intelligence] explode all trees<br>(machine learning):ti,ab,kw<br>MeSH descriptor: [Models, Statistical] this term only<br>MeSH descriptor: [Data Mining] this term only<br>MeSH descriptor: [Logistic Models] this term only<br>MeSH descriptor: [Computer Simulation] this term only<br>(Computer Simulation):ti,ab,kw<br>(predict*):ti<br>((risk predict* Near/1 (tool* or model*))):ti,ab,kw<br>((risk adjust* Near/1 (tool* or model*))):ti,ab,kw<br>#11 or #12 or #13 or #14 or #15 or #16 or #17 or #18 or #19 or #20 or #21<br>MeSH descriptor: [Length of Stay] explode all trees<br>((unplanned or unexpected or extended) NEAR/4 stay):ti,ab,kw<br>(care Near/1 days):ti,ab,kw	(MH "Risk Assessment")<br>TI ((risk N1 (assess* or analys* or adjust*)) or AB ((risk N1 (assess* or analys* or adjust*))<br>(MH "Severity of Illness Indices")<br>(MH "Predictive Value of Tests")<br>TI "Risk Adjustment" or AB "Risk Adjustment"<br>(MH "Risk Factors")<br>TI "risk predict* factor*" or AB "risk predict* factor*"<br>(MH "Electronic Health Records")<br>TI (electronic health record* or electronic medical record* or emr or ehr)<br>AB (electronic health record* or electronic medical record* or emr or ehr)<br>S1 OR S2 OR S3 OR S4 OR S5 OR S6 OR S7 OR S8 OR S9 OR S10<br>(MH "Regression")<br>(MH "Artificial Intelligence")<br>TI machine learning OR AB machine learning<br>(MH "Models, Statistical")<br>(MH "Data Mining")<br>TI logistic model* OR AB logistic model*<br>(MH "Computer Simulation")<br>TI computer simulation OR AB computer simulation<br>TI predict*<br>TI ((risk predict* n1 (tool* or model*))) OR AB ((risk predict* n1 (tool* or model*)))<br>TI ((risk adjust* n1 (tool* or model*))) OR AB ((risk predict* n1 (tool* or model*)))<br>S12 OR S13 OR S14 OR S15 OR S16 OR S17 OR S18 OR S19 OR S20 OR S21 OR S22<br>(MH "Length of Stay")<br>TI (((unplanned or unexpected or extended) n4 stay*)) OR AB (((unplanned

129263<br>29 25 or 26 or 27 or 28<br>129958		((length near/2 stay)):ti OR<br>((length near/2 stay)):kw<br>#23 or #24 or #25 or #26<br>#10 and #22 and #27	or unexpected or extended)<br>n4 stay*))<br>TI care n1 days OR AB care<br>n1 days<br>TI (length* n2 stay*)<br>S24 OR S25 OR S26 OR S27<br>S11 AND S23 AND S28

Table S3: Inclusion/exclusion criteria

	Inclusion	Exclusion
**Population**	Adults admitted to acute hospital care<br>Studies from OECD countries<br>General Medicine admissions<br>All Admissions (mixed Gen med and surgical)	Newborn/infants/age<15 yrs old<br>Maternity admissions<br>Paediatric admissions<br>Other single diagnostic groups<br>Only surgical admissions<br>Nursing home/community hospital/ICU/ED only populations<br>All day admissions <24 hrs
**Intervention**	Risk prediction tools/models<br>Studies reporting metrics of prediction or model predictive performance	Studies reporting only association metrics between dependant variable (LOS) and variables/features tested
**Outcomes**	Length of stay prediction in days/hours	LOS<24 hours/day admissions<br>Cumulative/aggregate measures of LOS<br>Studies reporting other outcomes like mortality/complications etc as a proxy for prolonged LOS

Table S4: List of data items extracted for each included study:

- Study Author
- Title
- Year of publication
- Lead authors contact details
- Country in which the study conducted
- Study funding sources
- Possible conflicts of interest for study authors
- Aim of study
- Study design
- Study Temporality
- Population description (from which study participants are drawn)
- Setting (include location and social context)
- Study Inclusion criteria
- Study Exclusion criteria
- Diagnostic groups
- Data Source DD

- Total number of participants
- Total number of admissions
- Study Duration (Years)
- Number of Candidate predictors
- Type of predictors used: 1) administrative, 2) demographic and anthropometric, 3) diagnosis (primary and secondary including comorbidities) and procedure types, 4) physical examination (biological and physiological parameters), 5) risk scores (e.g., Frailty Index, Charlson Comorbidity Index (CCI)), 6) admission characteristics, 7) hospital characteristics, 8) healthcare professional characteristics, 9) documentation and clinical notes and 10) medications.
- Timing of predictor measurement: Admission/Preadmission, During hospitalisation, Discharge/Post discharge
- Data analysis/modelling method used: classical statistical methods, machine learning or both
- Details of ML method used
- Method for selection of predictors for inclusion in multivariable modelling
- Method for selection of predictors during multivariable modelling like full model approach, backward or forward selection and criteria used (e.g., p-value, Akaike Information Criterion)
- Shrinkage of predictor weights or regression coefficients (e.g., no shrinkage, uniform shrinkage, penalized estimation)
- Handling of missing data: excluded/imputed/complete case analysis/other
- Transformation done of LOS. E.g., log/square root
- Hyperparameter tuning and selection methods
- Calibration (calibration plot, calibration slope, Hosmer-Lemeshaw test)
- Classification / Discrimination measures (e.g., AUROC, C index sensitivity, specificity, predictive values, net reclassification improvement) and whether a priori cut points were used
- Overall Measures/goodness of fit measures (MSE, RMSE, Brier score, R2 statistic)
- Method used for testing and optimising model performance: development dataset only (random split of data, resampling methods, e.g., bootstrap or cross-validation, none) or separate external validation (e.g., temporal, geographical, different setting, diff
- In case of poor validation, whether model was adjusted or updated (e.g., intercept recalibrated, predictor effects adjusted, or new predictors added)
- Was the model externally validated?
- Was there any report of implementation and change in clinical practice
- LOS measured as Continuous/Categorical
- Other outcomes
- Prolonged LOS cut off levels for definition of LOS
- Key conclusions of study authors: interpretation of presented models- useful for practice vs exploratory, comparison with other studies, strengths, and limitations
- References to other relevant studies
- Correspondence required for further study information

Table S5: Predictor variable categories with possible inclusion examples. BMI♢: Body Mass Index, TAC^: Transport Accident Commission

	Predictor Variable Category	Example inclusions
**1.**	Administrative	Type of insurance, Workcover/TAC^ etc
**2.**	Demographic and anthropometric	Age, sex, BMI♢, ethnicity, carer status/living situation
**3.**	Diagnosis (primary and secondary including comorbidities) and procedure types	Diabetes, Hypertension, COPD, ICD diagnostic codes etc
**4.**	Physical examination (biological and physiological parameters)	Serum haemoglobin, albumin, globulin, BSL etc

**5.**	Risk scores	Frailty Index, Charlson Comorbidity Index (CCI), American Surgical Association (ASA) Score
**6.**	Admission characteristics	Emergency/elective, from home/residential care
**7.**	Hospital characteristics	Rural vs urban, teaching, private/public etc
**8.**	Healthcare professional characteristics	Years of experience, volume of surgery, seniority
**9.**	Documentation and clinical notes	Structured and unstructured text from medical records
**10.**	Medications	Psychotropic drugs, insulin, opioid medications etc

Table S6a: Study characteristics of 39 included studies (Country, Study Temporality, Study design, Diagnostic Group, setting (include location and social context), Study Duration (Years))

Study Author, Year	Country study conducted	Study Temporality	Study design	Setting (include location and social context)	Data Source	Study Duration (Years)
			**Gen Medical**			
Barnes 2016	United States	retrospective	Cohort study	single, 36-bed medical unit in a large, mid-Atlantic academic medical center serving an urban population.	Medical records	3
Cournane 2015a	Ireland	retrospective	Cohort study	secondary referral centre in Dublin, Ireland	Administrative Data/Claims/Registry	12
Cournane 2015b	Ireland	retrospective	Cohort study	secondary referral centre in Dublin, Ireland	Administrative Data/Claims/Registry	12
Doctoroff 2020	United States	retrospective	Cohort study	US non-federal, short-term hospitals, including public hospitals, community hospitals, and academic medical centers	Administrative Data/Claims/Registry	1
Ferrao 2021	Other: Portugal	retrospective	Cohort study	Nonsurgical inpatients from a large public hospital in Portugal	Administrative Data/Claims/Registry	0.5
Launay 2014	Other: France	prospective	Cohort study	Angers University Hospital France	Administrative Data/Claims/Registry	1
Launay 2015	Other: France	prospective	Cohort study	ED in Angers University Hospital, France	Administrative Data/Claims/Registry	1
Launay 2018	Other: France	prospective	Cohort study	Angers University Hospital, France.	Administrative Data/Claims/Registry	1
Nguyen 2015	Australia	prospective	Cohort study	Large tertiary hospital Adelaide Australia	Medical records	0.5
Pilotto 2016	Other: Italy	prospective	Cohort study	20 geriatric units across Italy	Medical records	0.2
Romero-Ortuno 2012	Other: Ireland	retrospective	Cohort study	Largest tertiary teaching hospital Dublin, Ireland.	Administrative Data/Claims/Registry	11
Sander 2020	Germany	prospective	Cohort study	Major regional secondary care hospital ED RoMed Hospital of Rosenheim, Germany	Medical records	0.2
Symum 2020	United States	retrospective	Cohort study	general, teaching, and specialized hospitals network Tampa Bay region, Florida, USA.	Administrative Data/Claims/Registry	4.5
Yokokawa 2022	Japan	prospective	case control study	three general internal medicine wards in Chiba Prefecture, Japan	Medical records	2

All Admissions						
Baek 2018	Other: South Korea	retrospective	Cohort study	Tertiary general hospital in Seoul	Medical records	1
Bahrmann 2018	Germany	prospective	Cohort study	large tertiary care center Nuremberg, Germany	Medical records	0.6
Beaulieu-Jones 2021	United States	retrospective	Cohort study	public and private hospitals in the USA	Administrative Data/Claims/Registry	6
Belderrar 2017	Other: Algeria	retrospective	Case report	MIMIC-III - patients admitted to the Beth Israel Deaconess Medical Center in Boston, Massachusetts	Medical records	12
Chrusciel 2021	France	Retrospective	Cohort study	patients admitted to ED of the Centre Hospitalier de Troyes, a large rural French hospital	Medical Records	1
Gilbert 2018	UK	retrospective	Cohort study	Hospital Episode Statistics (HES) inpatient database of NHS London	Administrative Data/Claims/Registry	1
Grampurohit 2020	United States	retrospective	Cohort study	MIMIC 2 - patients admitted to the Beth Israel Deaconess Medical Center in Boston, Massachusetts	Medical records	7
Guerra 2015	Other: Portugal	prospective	Cohort study	Portuguese university hospital	Medical records	2
Harutyunyan 2019	United States	retrospective	Cohort study	MIMIC-III - patients admitted to the Beth Israel Deaconess Medical Center in Boston, Massachusetts	Medical records	12
Hilton 2020	United States	retrospective	Cohort study	tertiary academic medical centre in Cleveland Ohio	Medical records	7.5
Jaotomobo 2023	France	retrospective	Cohort study	public tertiary-care center with 3,400 beds and 2,000 physicians French Medico-Administrative database (PMSI)	Administrative Data/Claims/Registry	1
Lequertier 2021	Other: France	retrospective	Cohort study	6 hospitals of Lyon, France	Administrative Data/Claims/Registry	8
Levin 2021	United States	Cross sectional study	Cross sectional study	community hospital in Columbia	Medical records	1.5
Liu 2010	United States	retrospective	Cohort study	17 Northern California Kaiser Permanente Medical Care Program (NC-KPMCP) hospitals	Medical records	1.5
Liu 2019	United States	retrospective	Cohort study	large hospital system (community hospital and two tertiary/quaternary care hospitals with >2,000 beds and over 100,000 patient admissions annually) in the New York City metropolitan area.	Medical records	2
Malone 2018	United States	retrospective	Cohort study	Beth Israel Deaconess Medical Center, Boston MA	Medical records	11
Mcalister 2019	Canada	retrospective	Cohort study	Ontario province, Canada	Administrative Data/Claims/Registry	6
Monterde 2020	Other: Spain	retrospective	Cohort study	large public healthcare provider Catalan Institute of Health (ICS) Barcelona	Administrative Data/Claims/Registry	1
Ossai 2022	Australia	Retrospective	Cohort study	private acute teaching hospital	Administrative Data/Claims/Registry	5

Purushotham 2018	United States	retrospective	Cohort study	MIMIC-III - patients admitted to the Beth Israel Deaconess Medical Center in Boston, Massachusetts	Medical records	10
Rajkomar 2018	United States	retrospective	Cohort study	Hospitals run by University of California, San Francisco (UCSF) and the University of Chicago Medicine (UCM)	Medical records	7
Shin 2020	Japan	retrospective	Cohort study	data from over 500 acute care hospitals public and private across Japan	Administrative Data/Claims/Registry	2
Shukla 2018	United States	retrospective	Cohort study	MIMIC-III - patients admitted to the Beth Israel Deaconess Medical Center in Boston, Massachusetts	Medical records	11
Soong 2019	Singapore	retrospective	Cohort study	34 hospitals from nine countries across Europe, Australia, the UK and USA	Administrative Data/Claims/Registry	4
Xiongcai 2016	Australia	retrospective	Cohort study	All ED admissions to Sydney metropolitan hospital	Administrative Data/Claims/Registry	3.5

Table S6b: Study characteristics of 39 included studies (Sample size, Study Inclusion criteria, Study Exclusion criteria, Primary Outcome LOS definition, Timing of predictor measurement, Type of predictors used)

**Study Author, Year**	**Final Sample size**	**Study Inclusion criteria**	**Study Exclusion criteria**	**Primary Outcome LOS definition**	**Timing of predictor measurement:**	**Type of predictors used**
			**Gen Medical**			
Barnes 2016	8,852	all General medical ward patients	not specified	Probability of discharge by 2 pm or EOD	Admission; Other: daily at 7am for prediction for 2 pm dc on the same day	Administrative data; Demographics and anthropometric; Diagnoses and Medical history (includes Co-morbidities); Timing and frequency of admission; Other: patient flow data
Cournane 2015a	66,933	all emergency med admissions	nil	LOS > 5.1 d	Admission	Administrative data; Demographics and anthropometric; Diagnoses and Medical history (includes Co-morbidities); Physical examination: Biological and Physiological Parameters; Risk score EG CCI/CFI etc; Patient documentation and clinical notes
Cournane 2015b	66,933	all emergency General medical admissions	nil	LOS > 5.1 d	Admission	Administrative data; Demographics and anthropometric; Diagnoses and Medical history (includes Co-morbidities); Physical

						examination: Biological and Physiological Parameters; Risk score EG CCI/CFI etc; Timing and frequency of admission
Doctoroff 2020	29,97,249	adult >=18, medical hospitalization,	primary diagnosis = rehabilitation care, episodes with missing outcome (LOS), and predictor variables	LOS > 21 d	Admission	Administrative data; Demographics and anthropometric; Diagnoses and Medical history (includes Co-morbidities); Timing and frequency of admission
Ferrao 2021	5,089	all nonsurgical patients admitted to said hospital in the 6-month study period	not specified	LOS <=1,<br>LOS=2 to 7<br>LOS= 8 to 12<br>LOS Outliers >12	Throughout the hospital admission	Demographics and anthropometric; Diagnoses and Medical history (includes Co-morbidities); Physical examination: Biological and Physiological Parameters; Other: prescriptions and Medications
Launay 2014	1,254	Age>=75 yr., unplanned ED admission, non DNR	Discharge home from ED, Missing data	LOS > 13 d	Admission	Diagnoses and Medical history (include Co-morbidities); Risk score EG CCI/CFI etc; Other: BGA items
Launay 2015	9,33	hospitalization in acute care wards after an ED visit, age > 80 years willingness to participate in research.	participants with missing data	LOS >=13 d	Admission	Administrative data; Demographics and anthropometric; Diagnoses and Medical history (includes Co-morbidities); Risk score EG CCI/CFI etc; Other: Risk score: BGA 10 item
Launay 2018	1,117	hospitalized on acute medical care wards after an ED visit, age 65 years and over, and willing to participate in research.	pts who died during admission, pts with missing data	LOS>13 days<br>top tertile	Admission	Administrative data; Demographics and anthropometric; Diagnoses and Medical history (includes Co-morbidities); Risk score EG CCI/CFI etc; Other: Risk score: 10 item Brief geriatric index (BGA)
Nguyen 2015	752	adult patients admitted through an Acute Medical Unit	not specified	LOS > 3 d	Admission	Demographics and anthropometric; Physical examination: Biological and Physiological Parameters
Pilotto 2016	1,908	age >=65 yr, admitted to hospital with acute	as above, patients who died before 10 days	LOS >10 d	Admission	Risk score EG CCI/CFI etc; Other: MPI includes functional status (ADL),

		disease or relapse of chronic disease, ability to provide consent, availability of standardized CGA (comprehensive Geriatric Index) to calculate MPI (Multidimensional prognostic index)				cognitive status (SPMSQ), nutritional status (MNA), skin integrity (Exton Smith scale), comorbidity (cumulative illness rating scale), number of drugs, and cohabitation status.
Romero-Ortuno 2012	1,938	patients transferred to ED from Nursing home	non nursing home admissions	LOS>30 days	Admission	Demographics and anthropometric; Diagnoses and Medical history (includes Co-morbidities); Physical examination: Biological and Physiological Parameters; Risk score EG CCI/CFI etc; Other: risk score CCI
Sander 2020	1,117	medical patients admitted to ED	patients with missing data	LOS (continuous)	Admission	Physical examination: Biological and Physiological Parameters; Risk score EG CCI/CFI etc; Other: MTS, MEWS 1, MEWS 2, MEWS 3, CART, PEWS, WPSS
Symum 2020	10,93,177	Discharges with primary diagnosis ICD-9 code AMI, CHF, COPD, DB, and PN	not specified	Prolonged LOS >7 days (85th Percentile)	Admission, Other: post-discharge	Demographics and anthropometric; Diagnoses and Medical history (includes Co-morbidities); Timing and frequency of admission; Hospital Characteristics; Other: disease-specific features, socio-economic status
Yokokawa 2022	33	all gen medical ward patients	readmission from last 2 weeks	LOS>14 Days	Admission	Demographics and anthropometric; Diagnoses and Medical history (includes Co-morbidities); Risk score EG CCI/CFI etc
**All Admissions**						
Baek 2018	45,546	index admission,	unplanned readmission, lack of discharge date, day surgery	LOS (continuous) LOS>30 days	Throughout the hospital admission	Administrative data: Diagnoses and Medical history (includes Co-morbidities); Health care professional characteristics; Other: antibiotics

Bahrmann 2018	332	consecutive non-trauma patients aged >68, admitted to the ED	acute ST-elevation MI, planned elective coronary revascularization, hospitalization for unstable angina within the preceding 2 months, coronary-artery bypass grafting or percutaneous transluminal angioplasty within the preceding 3 months, renal failure requiring dialysis, trauma with suspected myocardial contusion, life expectancy <6 months, or if they did not consent to providing a blood sample for use by the research team	LOS (continuous)	Admission	Administrative data; Demographics and anthropometric; Diagnoses and Medical history (includes Co-morbidities); Physical examination: Biological and Physiological Parameters; Risk score EG CCI/CFI etc
Beaulieu-Jones 2021	4,28,96,026	all hospitalisations with LOS>1 day	hospitalization > mortality excluded from readmission cohort, and if mortality <7day, then excluded from LOS cohort	LOS > 7 days	Admission; Day 1-3;	Administrative data; Demographics and anthropometric; Diagnoses and Medical history (includes Co-morbidities); Timing and frequency of admission; Health care professional characteristics; Patient documentation and clinical notes; Other: EMR data
Belderrar 2017	26,897	adult pts >16 yrs admitted to ICU patients	missing predictive factors, patients >89 yo	High Hospital stay Outliers (HHSO) = geometric mean +2SD	Throughout the hospital admission	Administrative data; Demographics and anthropometric; Diagnoses and Medical history (includes Co-morbidities); Physical examination: Biological and Physiological Parameters; Hospital Characteristics; Patient documentation and clinical notes
Chrusciel 2021	5006	All ED admissions, with LOS >2 days	Non-ED adm with LOS<48 hours	LOS>=7 days	Admission	Demographics and anthropometric; Diagnoses and Medical

						history (includes Co-morbidities); Physical examination: Biological and Physiological Parameters; Timing and frequency of admission
Gilbert 2018	10,13,590	aged 75 years and older who were discharged from hospital between April 1, 2013, and March 31, 2015. The cohort was restricted to elective, non-elective, and day case admissions to NHS hospitals,	excluding mental health and community health providers	LOS> 10 days	Other: post discharge	Risk score EG CCI/CFI, Demographics and anthropometric
Grampurohit 2020	4,927	not specified	not specified	LOS (continuous)	Admission; Other: throughout the admission	Administrative data; Demographics and anthropometric; Diagnoses and Medical history (includes Co-morbidities); Physical examination: Biological and Physiological Parameters; Hospital Characteristics; Patient documentation and clinical notes; Other
Guerra 2015	682	aged>18 years, white, given an expected length of stay>24 hours, conscious, cooperative, and able to provide written informed consent.	Patients unable to perform HGS technique, pregnant women, individuals in isolation, those who were admitted for procedures that implied strict bed rest (e.g., biopsies) in which the study protocol evaluation could put them clinically at risk, and those with hemodynamic instability	LOS >=7 d	Admission	Physical examination: Biological and Physiological Parameters; Risk score EG CCI/CFI etc
Harutyunyan 2019	42,276	adult patients 18 and above, admitted to ICU	patients <18 years	LOS >=7 d	Admission; Day 1-3	Demographics and anthropometric; Physical examination: Biological and Physiological Parameters

Hilton 2020	14,85,880	all patients admitted to hospital	patients admitted for observation only	LOS >5 d	Admission; Other: Discharge	Demographics and anthropometric; Diagnoses and Medical history (includes Co-morbidities); Hospital Characteristics; Patient documentation and clinical notes
Jaotomobo 2023	118650	older than 18 years old and with a length of stay (LOS) > 24 hours	in-hospital mortalities and obstetrical stays.	LOS>14 days	Admission	demographics, diagnoses, health prof characteristics, admission characteristics
Lequertier 2021	5,51,684	admission to participating hospitals via Emergency or elective	<18 yrs, medical units with <100 stays in 8 yrs, stays <2 days, stays with erroneous info. and stays >31 days	LOS 0-13 days<br>LOS > 13 days	Other: post discharge	Administrative data; Demographics and anthropometric; Diagnoses and Medical history (includes Co-morbidities)
Levin 2021	12,470	all adult patients admitted to four hospital units (two medical, surgical and telemetry)	nil specific	Discharge probability of Same Day/Next day /following day	Throughout the hospital admission	Administrative data; Demographics and anthropometric; Diagnoses and Medical history (includes Co-morbidities); Physical examination: Biological and Physiological Parameters; Patient documentation and clinical notes; Other: interventions, consults, diet, clinical flags
Liu 2010	1,55,474	1)hospitalization began between January 1, 2002 through July 31, 2003 and October 1, 2003 through June 30,2005; (2) initial hospitalization occurred at a NC-KPMCP hospital (i.e., whether a hospitalization involved inter hospital transfer, the first hospital stay occurred within NC-KPMCP);(3) age>15 years at admission; and	age< 15, hospitalised for childbirth, and initial hospitalisation not in NC-KPMCP hospital	LOS (continuous)<br>LOS outliers >7, 10 and 15 days	Preadmission; Admission	Administrative data: Diagnoses and Medical history (includes Co-morbidities); Physical examination: Biological and Physiological Parameters; Other: LAPS and COPS automated scores

		(4)hospitalization was not for childbirth.				
Liu 2019	1,23,641	adult (18 years) inpatient hospital discharges	nil	LOS (continuous)	Admission	Administrative data; Demographics and anthropometric; Diagnoses and Medical history (includes Co-morbidities); Risk score EG CCI/CFI etc
Malone 2018	17,869	all pts admitted to ICU	not specified	LOS (continuous)	Day 1-3; Other: 48 hours after admission to ICU	Administrative data; Demographics and anthropometric; Diagnoses and Medical history (includes Co-morbidities); Physical examination: Biological and Physiological Parameters; Patient documentation and clinical notes
Mcalister 2019	4,52,785	age>75 yrs, at least one urgent non-psychiatric hospitalisation between 2004 and 2010. *Included patients in long term care facility	excluded mental health and community provider admissions	LOS > 10 d	Admission	Diagnoses and Medical history (includes Co-morbidities); Risk score EG CCI/CFI etc
Monterde 2020	1,56,459	all discharges (home or dead) following standard hospital stays. Multiple admissions included	hospital stays for major ambulatory surgical interventions	LOS > 14 d	Other: post discharge	Administrative data; Diagnoses and Medical history (includes Co-morbidities); Risk score EG CCI/CFI etc; Other: CCI, Elixhauser, Queralt Indices
Ossai 2022	91468	all planned elective admissions	nil	ELOHS (tertile for DRG)	Admission	Demographics, risk scores, health professional and admission characteristics.
Purushotham 2018	38,425	Adults (>15 yr) admitted to ICU, data extracted only for the first admission to ICU	subsequent admissions to ICU	LOS (continuous)	Admission; Day 1-3; Other: first 24 hrs and 48 hr data	Demographics and anthropometric; Diagnoses and Medical history (includes Co-morbidities); Physical examination: Biological and Physiological Parameters; Risk score EG CCI/CFI etc; Other: SAPS II (Simplified Acute Physiology Score) and SOFA (Sepsis-related Organ failure assessment)

Rajkomar 2018	2,16,221	adult patients hospitalized for at least 24 h.	not specified	LOS > 7 d	Admission; Other: at 24 hours	Demographics and anthropometric; Diagnoses and Medical history (includes Co-morbidities); Physical examination: Biological and Physiological Parameters; Patient documentation and clinical notes
Shin 2020	26,71,749	non-maternal inpatients aged >18 yrs	special purpose hospitalization e.g., chemo, clinical trials, and LOS<1 day	LOS (continuous)	Admission; Other: Discharge	Diagnoses and Medical history (include Co-morbidities)
Shukla 2018	53,211	ns	LOS<48 hours	LOS (continuous)	Throughout the hospital admission	Physical examination: Biological and Physiological Parameters
Soong 2019	13,66,187	>=75 yrs. elective or non-elective hospital admission of 24 hours or more.	age, sex, or length of stay was recorded as missing or invalid, or the admission was planned and the patient discharged home on the same day, or the admission was unplanned, but no procedure was undertaken and the patient went home after the recorded length of stay less than 2 days.	upper quartile of hospital LOS for country	Other: post discharge	Administrative data; Demographics and anthropometric; Diagnoses and Medical history (includes Co-morbidities)
Xiongcai 2016	32, 634	All ED admissions	patients with unlinked data	probability of being in hospital/ at home/dead	Admission; Day 1-3; Day 4-7	Demographics and anthropometric; Diagnoses and Medical history (includes Co-morbidities); Physical examination: Biological and Physiological Parameters; Timing and frequency of admission

Table S6c: Study characteristics of 39 included studies (Predictive modelling methods and Analytical Pipeline)

Study Author, Year	Data analysis/ modelling method used	Number of variables	Imputation of Missing Data	Method for selection of predictors for inclusion in multivariable modelling	Method for selection of predictors during multivariable modelling	Transformation done of LOS?	Hyperparameter tuning and selection?	Method used for testing and optimising model performance
**Gen Medical**								

Barnes 2016	Both	11	not specified	all candidate predictors	not specified	no	not specified	random split of data, bootstrapping, cross validation
Cournane 2015a	Classical statistical approach	15	not specified	pre-selection based on unadjusted association with the outcome like univariable modelling	p<0.10	not specified	n/a	used robust standard errors for parameter estimates
Cournane 2015b	Classical statistical approach	41	not specified	pre-selection based on unadjusted association with the outcome like univariable modelling	p<0.10	not specified	n/a	sensitivity analysis done by including the long stay patients
Doctoroff 2020	Classical statistical approach	5	not specified	all candidate predictors	p<0.05	no	n/a	random data split, sensitivity analysis
Ferrao 2021	Both	1001	Partial, if missing data rates <50%. Features excluded if >50% missing data	all candidate predictors	forward-backward stepwise selection for multilinear regression (starting from empty feature sets, maximum inclusion and minimum removal p-value thresholds set to 0.05 and 0.1, respectively, with an infinite number of iterations)	regression models were developed using log-transformed data	not specified	5-fold cross-validation. temporal analysis
Launay 2014	Classical statistical approach	5	not specified	pre-selection based on unadjusted association with the outcome like	not specified	no	n/a	not specified

				univariable modelling				
Launay 2015	Machine Learning Approach	15	not specified	all candidate predictors	not specified	no	not specified	random split of data: train and test
Launay 2018	Machine Learning Approach	15	patients with missing data excluded	pre-selection based on unadjusted association with the outcome like univariable modelling	not specified	no	not specified	train and test
Nguyen 2015	Classical statistical approach	7	not specified	pre-selection based on unadjusted association with the outcome like univariable modelling	not specified	not specified	n/a	not specified
Pilotto 2016	Classical statistical approach	9	not specified	all candidate predictors	not specified	no	n/a	not specified
Romero-Ortuno 2012	Classical statistical approach	17	not specified	all candidate predictors	not specified	not specified	n/a	not specified
Sander 2020	Classical statistical approach	5	No, patients with missing data were excluded	all candidate predictors	not specified	not specified	n/a	not specified
Symum 2020	Machine Learning Approach	17	Partial, less than 15% of the records were missing, the mean or median value replaced the blanks for the continuous and ordinal feature respectively. over 15% missing cases, we excluded the attributed feature	all candidate predictors	chi-square filtering at p-value <0.05 and SVM wrapper based algorithm limited to 200 iterations	no	not specified	random split of data, SMOTE (oversampling) to address class imbalance, data pre-processing to address complexities, used multiple metrics and aggregate ranking method

Yokokawa 2022	Classical statistical approach	6	No, patients with missing data were excluded	all candidate predictors	not specified	no	na	k fold cross validation
**All Admissions**								
Baek 2018	Classical statistical approach	5	not specified	pre-selection based on unadjusted association with the outcome like univariable modelling	not specified	not specified	n/a	dev: random split of data 80/20
Bahrmann 2018	Classical statistical approach	2	not specified	all candidate predictors	not specified	not specified	n/a	not specified
Beaulieu-Jones 2021	Both	21	not specified	all candidate predictors	not specified	not specified	hyperparameter values provided in Supp Table 7. unclear how these were selected	cross validation
Belderrar 2017	Machine Learning Approach	28	not specified	all candidate predictors	not specified	Yes - z score standardization, min/max normalization	not specified	random data split 80:20
Chrusciel 2021	Machine Learning Approach	11	not specified	all candidate predictors	not specified	not specified	yes	3-fold cross validation, hyper parameter optimisation
Gilbert 2018	Classical statistical approach	18	not specified	all candidate predictors	not specified	not specified	n/a	cross-validation, temporal validation, sensitivity analysis
Grampurohit 2020	Machine Learning Approach	22	not specified	all candidate predictors	not specified	not specified	not specified	Random split
Guerra 2015	Classical statistical approach	8	not specified	all candidate predictors	AIC	no	n/a	not specified
Harutyunyan 2019	Both	714	Yes, missing values using the most recent measurement value if it	all candidate predictors	not specified	no	number of memory cells in LSTM layers, the dropout	random split, bootstrapping

			exists and a pre-specified “normal” value otherwise				rate, and whether to use one or two LSTM layers	
Hilton 2020	Both	8	Yes, indirectly by using algorithm that can explicitly account for missing data	all candidate predictors	not specified	not specified	Bayesian hyperparameter optimization	Random split, int validation, cross-validation, early stopping at 200 iterations,
Jaotomobo 2023	Machine Learning Approach	27	no	all candidate predictors	not specified	no	not specified	stochastic gradient descent
Lequertier 2021	Machine Learning Approach	110	not specified	all candidate predictors	not specified	no	gradient descent: Scaled Exponential Linear Unit (SELU) activations	5 fold cross validation
Levin 2021	Machine Learning Approach	110	not specified	all candidate predictors	full model approach	not specified	not specified	cross validation
Liu 2010	Classical statistical approach	7	not specified	all candidate predictors	not specified	yes	n/a	not specified
Liu 2019	Classical statistical approach	9	not specified	all candidate predictors	not specified	no	n/a	not specified
Malone 2018	Classical statistical approach	26	No, missing data representations were used as a part of the modelling techique	all candidate predictors	not specified	not specified	n/a	regularization parameter
Mcalister 2019	Classical statistical approach	2	not specified	all candidate predictors	full model approach	not specified	n/a	external validation study
Monterde 2020	Classical statistical approach	9	not specified	all candidate predictors	AIC	not specified	n/a	not specified
Ossai 2022	Machine Learning Approach	99	no	all candidate predictors	SMOTE + Recursive Feature Elimination	no	not specified	cross validation and Extra tree classifier

Purushotham 2018	Machine Learning Approach	136	Yes, forward and backward imputation for partial missing and mean imputation for complete missing	all candidate predictors	AIC, p value	no	not specified	cross validation, random split
Rajkomar 2018	Machine Learning Approach	28	not specified	all candidate predictors	not specified	not specified	hyperparameters were found via a Gaussian-process-based hyperparameter search on each dataset validation performance: listed in the supplementary material. Hyperparameters were tuned automatically using Google Vizier10 over thousands of experiments	random split, bootstrapping
Shin 2020	Classical statistical approach	3	not specified	all candidate predictors	not specified	not specified	not specified	random split, cross validations, bootstrapping
Shukla 2018	Both	12	No, patients with missing data were excluded	all candidate predictors	not specified	yes - log transformed	not specified	random split, bootstrap and cross-validation
Soong 2019	Classical statistical approach	2	No, patients with missing data were excluded	all candidate predictors	not specified	no	n/a	external validation,

Xiongcai 2016	Machine Learning Approach	64	Yes, Bayesian network model-based imputation approach	all candidate predictors; Other: correlation-based feature selection algorithm	not specified	not specified	not specified	random split, temporal validation

Table S7: Performance measures used in 39 included Studies.

Study Author, Year	Calibration	Classification / Discrimination measures	Overall Measures/goodness of fit measures
Barnes 2016	Nil	Youden's index, Sensitivity, Specificity	Nil
Cournane 2015a	HL goodness of fit test	AUROC	Nil
Cournane 2015b	HL goodness of fit test	AUROC	Nil
Doctoroff 2020	Nil	AUROC, C statistic	Nil
Ferrao 2021	Nil	AUROC	Accuracy, Precision, Recall, F1 score, RMSE, MAE, MAPE
Launay 2014	Nil	Sensitivity/spec/PPV/NPV/AUC	Nil
Launay 2015	Nil	Sensitivity, Specificity, PPV, NPV, LR, AUROC	Nil
Launay 2018	Nil	AUROC, Sens/spec/PPV/NPV	Nil
Nguyen 2015	Nil	NRI, C-statistic/C-index	Nil
Pilotto 2016	Nil	C-statistic/C-index	Nil
Romero-Ortuno 2012	Nil	Chaids model classification tree	Nil
Sander 2020	Nil	AUROC	Nil
Symum 2020	Nil	sensitivity, specificity, AUC	Nil
Yokokawa 2022	calibration plots	AUC	Nil
Baek 2018	Nil	classification accuracy	MAE
Bahrmann 2018	calibration plots	AIC	Nil
Beaulieu-Jones 2021	Nil	AUROC	Nil
Belderrar 2017	Nil	Pred (q)% (>= 75)	MMRE % (<=25)
Chrusciel 2021	precision	accuracy, recall, specificity	Fi score
Gilbert 2018	Nil	C statistic	Nil
Grampurohit 2020	Nil	Nil	MAE
Guerra 2015	Nil	AIC	Nil
Harutyunyan 2019	calibration plots	Cohen's Kappa score	Mean absolute difference (MAD)
Hilton 2020	calibration plots	AUROC, AUPRC (precision-recall curve), Brier score loss.	accuracy, and RMSE
Jaotomobo 2023	nil	AUC	nil
Lequertier 2021	Nil	Cohen's Kappa score	accuracy
Levin 2021	Nil	AUROC, sensitivity, specificity,	accuracy
Liu 2010	Nil	Nil	MSE, Accuracy
Liu 2019	Likelihood ratio index measures the proportionate reduction in the log-likelihood due to	Nil	Nil

	inclusion of regressors		
Malone 2018	Nil	AUROC	MAE
Mcalister 2019	Nil	C-statistic/C-index	Nil
Monterde 2020	Nil	AUROC, AIC, BIC, Brier's score	Nil
Ossai 2022	precision, Calibration plots	AUC, accuracy, recall	f1 score
Purushotham 2018	calibration plots	Nil	MSE
Rajkomar 2018	calibration plots	AUROC	Nil
Shin 2020	Nil	C-statistic/C-index	explained variance
Shukla 2018	Nil	AUROC, AUPRC (precision-recall curve)	Median AE, explained variance (EV) score
Soong 2019	mcali	AUROC	Nil
Xiongcai 2016	Nil	PPV, NPV, AUC,	accuracy

Table S8: Predictors included in the All-Admissions LOS models included in the systematic review (n=45)

Study	Type of final model	Input Variables	Outcome	Name of Data analysis/modelling method used DD
Baek 2018 (1)	Internal Validation	diagnosis frequency, insurance type (health insurance = 1), severity (y = 1), surgery frequency, transfer frequency	LOS Pred (continuous)	Multivariable Logistic Regression
Baek 2018 (2)			LOS long-term (>30 days)	Random forest method (ML)
Bahrmann 2018 (1)	Development	Barthel Index (BI) (Adjusted- age, sex, CCI)	LOS (continuous)	Multivariable Linear Regression
Bahrmann 2018 (2)		Charlson Co-morbidity index (CCI) (Adjusted- age, sex, BI)		
Beaulieu-Jones 2021 (1)	Development	demographic, admission characteristics, and all charges day 1 data	LOS > 7 days	stacked recurrent neural network (gated recurrent unit (GRU))
Beaulieu-Jones 2021 (2)	Temporal Validation			
Belderrar 2017	Internal Validation	Admission type, admitting unit/location, age, chart events, Comorbidities, Diagnosis, discharge location, ethnicity, height/weight, input CareVue: observations, input MetaVision imaging, insurance, Lab events, language, marital status, medication-related order entries, microbiology events, output fluids, procedural terminology, Religion Sex	High hospital LOS outliers (geometric mean = 2 SD)	FRBFN (fuzzy radial basis function networks)
Chrusciel 2021	Development	i) personal information such as age, gender and zip/postal code, ii) context information such as entry date, LOS at the ED, triage (CCMU and GEMSA) codes, iii) ICD-10 primary diagnosis code and iv) unstructured information such as the UMLS concepts extracted from the text documents uploaded during the stay at the ED.	LOS>=7days	Random forest method (ML)
Gilbert 2018	Internal Validation	Age, Sex, CCI HFRS (Hospital Frailty Risk Score) high/intermediate risk, socio economic index	LOS>10 days	Multivariable Logistic Regression
Grampurohit 2020	Development	Admission source, Admission type, Age, BMI, Care givers, Care units, Ethnicity, First diagnosis on admission, First procedure on admission, Gender Hospital admission ID, Imaging reports, Insurance, IV meds, Marital status, non-IV meds,	LOS (continuous)	Ridge regression

		Notes, Number of diagnoses on admission, Number of Micro labs/Number of lab tests, Orders Religion, SOFA first score		
Guerra 2015	Development	PG-SGA (Mod/suspected and severe) (adjusted for age, education, Katz index and CCI)	LOS >=7 d	Cox Proportional hazards regression model
Guerra 2015		NRS-2002 (at risk of undernutrition) (adjusted for age, education, Katz index and CCI)		
Harutyunyan 2019	Internal Validation	Observations: temp, HR, RR, SaO2, BP, FiO2, pH Anthropometric data, Glasgow Coma scale	LOS >7 days	Channel-wise LSTM + deep supervision
Harutyunyan 2019			LOS (continuous)	
Hilton 2020	Internal Validation	24 hr meds, Admission source, and type, age, early admission to ICU, insurance, last admission LOS, primary diagnosis code	LOS>5 days	Gradient Boosting Machines GBM based methods
Jaotomobo 2023	Development	age, gender, funder status, ICD10 comorbidity codes, patient origin (home or other hospital institution), hospitalization via emergency departments, destination after hospital discharge, hospitalization via emergency departments in the previous 6 months.	LOS>14 days	Gradient Boosting Machines GBM based methods
Lequertier 2021	Internal Validation	Age, Associated Diagnoses, Discharge date and time, Entry date and time, Gender, Hospital Medical Procedures, Medical Unit ID, Medical Unit type, Mode of entry, Principal Diagnosis, RUM ID	LOS 0-13 days LOS > 13 days	FFNN (Feed Forward Neural Network) with embeddings
Levin 2021 (1)	Internal Validation	Administrative, Clinical flags (correlates of psycho-social determinants), consults, Demographics, diagnostics (count of tests), diet, interventions, Medications (count of meds Oral/IV), monitoring outputs, rehabilitation (mobility scale) Temporal Variables: Elapsed hospital and unit LOS	LOS < 1 day (Same Day Discharge)	Supervised ML
Levin 2021 (2)			LOS < 2 days (Next Day Discharge)	Supervised ML
Liu 2019 (1)	Development	VW age-co-morbidity	LOS > 5 days	Multivariable Logistic Regression
Liu 2019 (2)	Development	APR-DRG 3M Severity of Illness		
Liu 2019 (3)	Development	APR-DRG 3M Risk of Mortality		
Liu 2010	Development	Admission diagnosis, Admission shift, Age, Co-morbidity Point score, Day of week, Laboratory Acute Physiology score, Sex	LOS (continuous)	OLS linear regression
Malone 2018	Internal Validation	Capillary refill rate, Diastolic blood pressure, ethnicity, Fraction inspired oxygen, Glasgow coma scale eye opening, Glasgow coma scale motor response, Glasgow coma scale total, Glasgow coma scale verbal response, Glucose, Heart Rate, Height, MARITAL STATUS, Mean blood pressure, EMR Notes DISCHARGE SUMMARY, ECG, ECHO, NURSING, RADIOLOGY, RESPIRATORY, Oxygen saturation, pH, Respiratory rate, Systolic blood pressure, Temperature, Weight	LOS (continuous) time series data only	Ridge regression
Malone 2018			LOS (continuous) all data	
McAlister 2019 (1)	External Validation	Hospital Frailty Risk Score (HFRS) unadjusted	LOS > 10 d	Multivariable Logistic Regression
McAlister 2019 (2)	External Validation	Hospital Frailty Risk Score (HFRS) adjusted		
Monterde 2020 (1)	Development	Queralt Diagnoses (pre-existing comorbidities only)	LOS >14 days	Multivariable Logistic Regression
Monterde 2020 (2)	Development	APR-DRG Severity of Illness		

Monterde 2020 (3)	Development	APR-DRG Risk of Mortality		
Monterde 2020 (4)	Development	All Queralt Diagnoses		
Ossai 2023	Development	10 features: visiting medical officer (VMO) specialty, patient age, patient gender, admission category (ADC), admission type, patient care class, Charlson Score, socioeconomic status (SES), and distance to hospital (DTH).	ELOHS (tertile for DRG)	Machine Learning: SMOTE + Recursive Feature Elimination with Cross-Validation (RFECV) + Extra Tree Classifier (ETC)
Purushottam 2018	Internal Validation	136 raw features from 5 relational tables: Admission type, age, AIDS, Bilirubin, Blood cancers, GCS, HR, Potassium, Metastatic cancer, Sodium, PaOo2/FiO2, SAPS-II scores, SBP, Serum bicarb level, Serum urea, Nitrogen level, Temp, Urine output, WBC count	LOS (continuous)	MMDL (multi modal deep learning)
Purushottam 2018	Internal Validation		LOS (continuous)	
Purushottam 2018	Development		LOS (continuous)	
Rajkomar 2018 (1) Hospital A	Internal Validation	admission source, age, gender, Hierarchical condition categories, hospital service, lab tests and hospital data at 24 hours,	LOS >7 days	Deep Learning
Rajkomar 2018 (2) Hospital B	Internal Validation			Deep Learning
Shin 2020	Internal Validation	Charlson comorbidities, Elixhauser Comorbidities, Gagne's comorbidities	LOS (continuous)	GLM with gamma distribution
Shukla 2018	Internal Validation	glucose, pH, SPO2, HR, RR, BP, Temp, urine output, FiO2	LOS (continuous)	Interpolation and Prediction network
Soong 2019 (1)	Development	BOTH Foster and Elixhauser (elective)	Upper quartile of LOS specific to country	Multivariable Logistic Regression
Soong 2019 (2)	Development	BOTH Foster and Elixhauser (Non-elective)		Multivariable Logistic Regression
Soong 2019 (3)	External Validation	Dr Foster Frailty Score (Elective)		Multivariable Logistic Regression
Soong 2019 (4)	External Validation	Dr Foster Frailty Score (Non-Elective)		Multivariable Logistic Regression
Xiongcai 2016	Internal Validation	Age, cancer, Day of week, days since last event (lab test etc), Elapsed LOS (current Admission), gender, marital code, mental co-morbidity, mode of arrival to ED, no. of days since last admission, Pathology tests at admission, time of day, total days in hospital in last 12/12, triage code, ward	LOS < 1 day (Same Day Discharge)	Machine Learning

Table S9: Most frequently used variables in risk prediction of prolonged LOS in GenMed studies (n=14)

**Input Variables (Predictors)**	**Frequency of inclusion in LOS risk prediction studies (n=14)**	
**Risk scores**<br>Brief Geriatric Assessment tool (BGA)<br>CCI<br>Simple Clinical Score (SCS) (comorbidities DM/Stroke/mental status/ECG)<br>Disabling score<br>Exton Smith scale (pressure injury risk)<br>illness Severity Index<br>Multidimensional prognostic Index (MPI)<br>Manchester triage scores<br>Nutritional status (MNA)	**12**	**86%**

Risk group (illness severity score)<br>Triage Group<br>mental status Questionnaire		
**Diagnoses (Primary/Secondary including comorbidities) and Procedure types**<br>>4 chronic diseases<br>Infectious disease: Influenza, Bacterial Pneumonia, Pneumoconiosis<br>Chronic Liver disease<br>Comorbidity: Coagulation disorders<br>Comorbidity: Fluid and electrolyte balance<br>Comorbidity: paralysis<br>Comorbidity: weight loss<br>Disabling score (diagnosis based)<br>Major diagnostic categories (MDC) nervous system disease, respiratory disease, Cardiovascular, Gastroenterology, Neurology<br>Reason for admission: Neuropsychiatric diseases, respiratory diseases	**11**	**79%**
**Demographic and Anthropometric**<br>Age,<br>Sex,<br>Age ≥ 85 years<br>Cohabitation status: living alone<br>Living situation: Home/ Institution/Nursing home	**10**	**71%**
**Physical examination (biological and physiological parameters)**<br>Allergies<br>AVPU score (alert/voice/pain/unresponsive)<br>Lab results- hematocrit, albumin, potassium, sodium, urea, WCC, cardiac troponin<br>Number of Tests/Imaging requests/medical and nursing procedures<br>Observation status- RR, SaO2, HR, Temperature, BP, DBP, SBP	**6**	**43%**
**Medications**<br>≥ 5 drugs per day<br>AND polypharmacy<br>Use of psychoactive drugs<br>No of Drugs on admission	**5**	**36%**
**Admission characteristics**<br>Day of the week: Monday, Friday, Saturday, Sunday<br>Disposition: Health Facility<br>Elapsed LOS	**2**	**14%**
**Healthcare professional characteristics**<br>consultant	**3**	**21%**
**Administrative**<br>Payer class-Uninsured	**1**	**7%**

Table S10: Abbreviations

**Abbreviations**	
LOS	Length of Stay
CCI	Charlson Comorbidity Index
FI	Frailty Index
ML	Machine Learning
AUROC	Area under Receiver Operating Curve
MSE	Mean Square Error
RMSE	Root Mean Square Error
CFS	Clinical Frailty Score
APR DRG	All Patient Refined

NRS	Nutritional Risk Score
MUST	Malnutrition Universal Screening Tool
SGA	Subjective Global Assessment
BMI	Body Mass Index
NRI	Net Reclassification Index
PPV	Positive Predictive Value
NPV	Negative Predictive Value
AIC	Akaike's Information Criterion
RSI	Risk Stratification Index
MAE	Mean Absolute Error
REML	Random-Effect Model Logistic
NC-KPMCP	North Carolina – Kaiser Permanente Medical Care Program
MIMIC	Medical Information Mart for Intensive Care

Table S11 Regression-based Egger test for small-study effects – All Admissions

Regression-based Egger test for small-study effects

Random-effects model

Method: REML

H0: beta1	0; no small-study effects
beta1	7.30
SE of beta1	11.806
z	0.62
Prob > |z|	0.5364

Table S12: Risk of Bias assessment of General Medicine studies using PROBAST tool (n=15). Dev: Development only (includes models with Internal Validation), Val: Studies with External Validation

	TYPE OF PREDICTION MODEL	PARTICIPANT RISK OF BIAS	PARTICIPANT APPLICABILITY	PREDICTOR RISK OF BIAS	PREDICTOR APPLICABILITY	OUTCOME RISK OF BIAS	OUTCOME APPLICABILITY	ANALYSIS OF RISK OF BIAS	OVERALL RISK OF BIAS	OVERALL APPLICABILITY CONCERN
**Barnes 2016**	**Development**	+	+	+	+	+	+	−	−	+
**Cournane 2015**	**Development**	−	−	+	+	+	+	−	−	−
**Cournane 2015**	**Development**	−	−	+	+	+	+	−	−	−
**Doctoroff 2020**	**Development**	+	+	+	+	+	+	−	−	+
**Ferrao 2021**	**Development**	?	?	+	+	+	+	−	−	?
**Launay 2014**	**Development**	+	+	+	+	+	+	−	−	+
**Launay 2015**	**Development**	+	+	+	+	+	+	−	−	+
**Launay 2018**	**Development**	+	+	+	+	+	+	−	−	+
**Nguyen 2015**	**Development**	+	+	+	+	+	+	−	−	+

**Pilotto 2016**	**Development**	+	+	+	+	+	+	−	−	+
**Romero-Ortuno 2012**	**Development**	+	+	+	+	+	+	−	−	+
**Sander 2020**	**Development**	+	+	+	+	+	+	−	−	+
**Symum 2020**	**Development**	?	?	+	+	+	+	−	−	?
**Yokokawa 2022**	**Development**	+	+	+	+	+	+	−	−	+

KEY: Low ROB + **Unclear ROB** ? **High ROB** −

Table S13: Study population and characteristics of the models included in meta-analysis of all admissions LOS prediction models (n=4)

**Study**	**Total Sample size**	**Predictors**	**Age**	**Prediction Analysis methods**	**AUROC values (95% CI)**	**Calibration metric**
Soong 2019 (3)	1,067,139	Age, Gender, Country, Elixhauser score, number of previous admissions, Dr Foster Frailty Score (Elective)	>75 yrs.	multivariable logistic regression model adjusted for age, gender, country	0.676 (0.675, 0.676)	HL goodness of fit test
Soong 2019 (4)	6,128,811	Age, Gender Country, Elixhauser score, number of previous admissions, Dr Foster Frailty Score (Non-Elective)	>75 yrs.		0.677 (0.676, 0.677)	HL goodness of fit test
McAlister 2019 (1)	4,52,785	Hospital Frailty Risk Score (HFRS) unadjusted	83.0±5.6	Logistic regression between HFRS and LOS>10 days	0.705 (0.703, 0.706)	Nil
McAlister 2019 (2)	4,52,785	Hospital Frailty Risk Score (HFRS) adjusted	83.0±5.6		0.723 (0.722, 0.724)	Nil

Figure 1 Frequency of categories of predictors used in General Medicine vs All Admissions models.

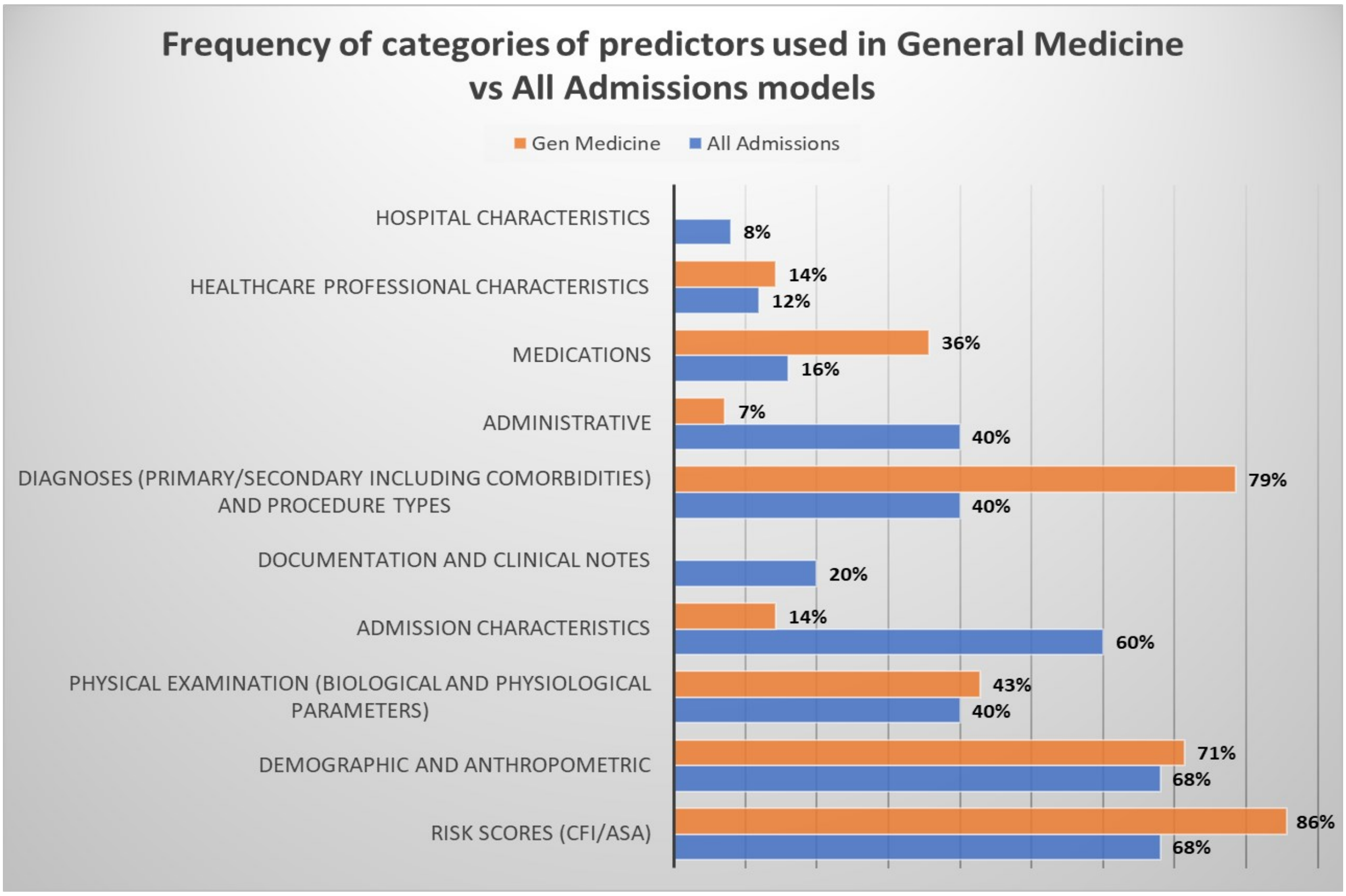

Figure 2: Frequency of LOS prediction model performance metrics reported in General Medicine LOS prediction models. (n=30)

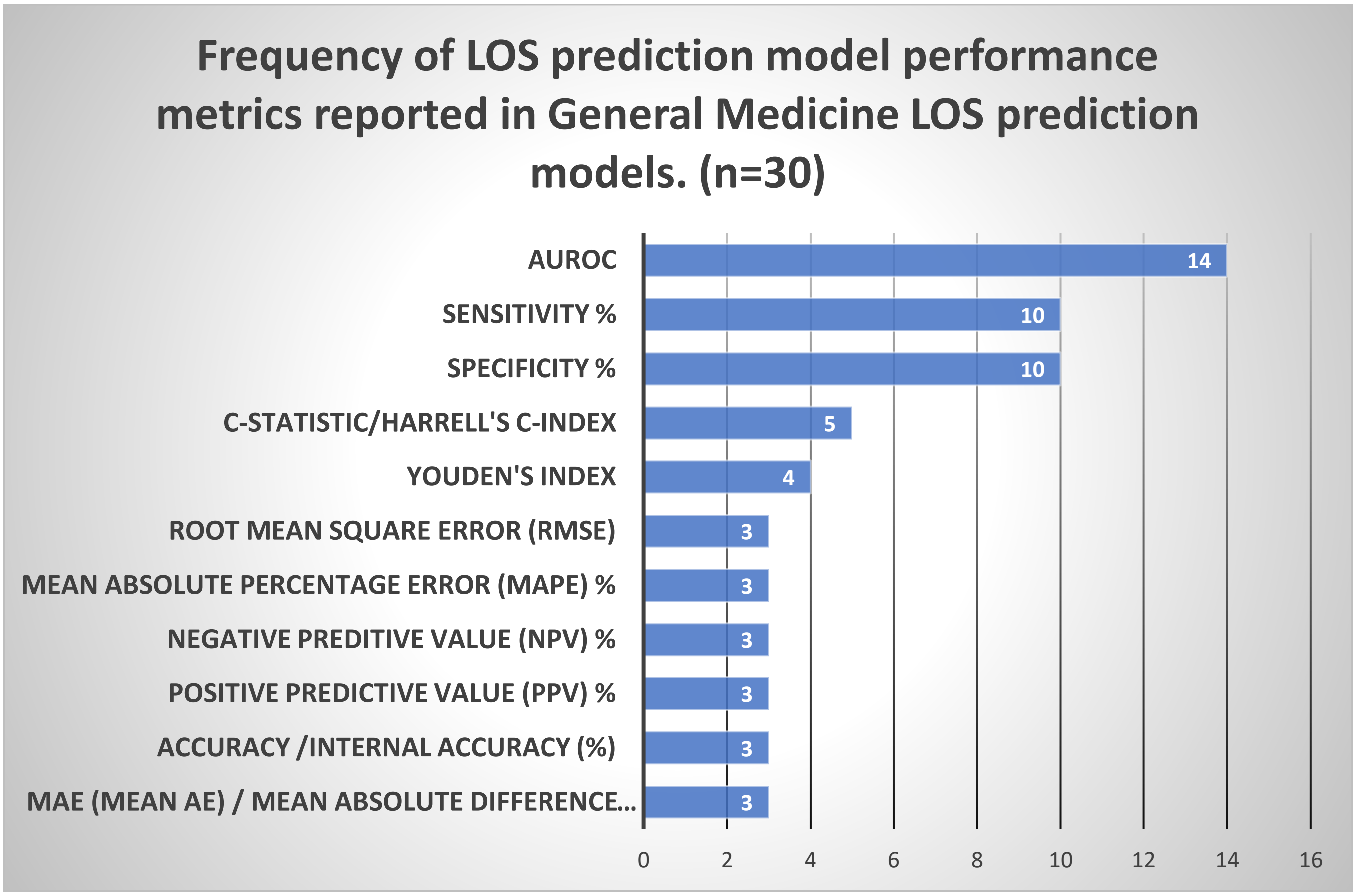

The following performance metrics were used less than 3 times and are not represented in the figure: Calibration slope/intercept, likelihood ratio index, F1 score, Recall, Precision, Hosmer-Lemeshaw Goodness of fit Tjur and Mcfadden's value, model adequacy, overall correct classification and Net Reclassification Index.
